# Decoding the human PBMC isonome: isoform-level resolution with single-cell long-read transcriptomics

**DOI:** 10.3389/fgene.2026.1782221

**Published:** 2026-05-28

**Authors:** Patricia Hayes Doyle, Madeline L. Page, J. Anthony Brandon, Bernardo Aguzzoli Heberle, Brendan J. White, Ann Marie Stowe, Mark T. W. Ebbert

**Affiliations:** 1 Sanders-Brown Center on Aging, University of Kentucky, Lexington, KY, United States; 2 Department of Neuroscience, College of Medicine, University of Kentucky, Lexington, KY, United States; 3 Division of Biomedical Informatics, Department of Internal Medicine, College of Medicine, University of Kentucky, Lexington, KY, United States; 4 Department of Neurology, College of Medicine, University of Kentucky, Lexington, KY, United States

**Keywords:** long-read single-cell RNA sequencing, Oxford Nanopore Technologies (ONT), PIPseq, PBMCs, RNA isoforms, isoform discovery, alternative splicing

## Abstract

Long-read single-cell RNA sequencing provides an opportunity to understand human health and disease at a level difficult to resolve with bulk or short-read methods. This approach enables isoform-level investigation of cellular diversity and disease mechanisms and definition of cell-types, rather than using genes alone. Using a modified, microfluidic-free PIPseq workflow and computational pipeline adapted for Oxford Nanopore long-read sequencing, we generated the largest long-read single-cell dataset of human peripheral blood mononuclear cells (PBMCs) from a single donor to date, the first with sufficient cell numbers to detect megakaryocytes. This study profiled isoform usage across immune cells, integrating marker expression and isoform discovery. We identified 126 novel isoforms from known and new genes, several with distinct cell-type-specific patterns, and characterized marker gene isoform expression across cell-types. Non-canonical protein-coding variants of *GZMB* and *CD3G* were enriched in unexpected cell-types, including megakaryocytes and monocyte-derived populations. We also discovered novel transcripts from *CMC1* and *LYAR* with cell-type-specific signatures that were also the predominantly expressed transcript within the gene. This study expands the versatility of long-read single-cell studies to not only relay changes in isoform signatures, but to position them within the functional context of the biology they impact. These results demonstrate the power of long-read single-cell sequencing for mapping the isoform landscape—the isonome—across tissues and disease contexts.

## Introduction

Various genetic and genomic approaches, including DNA and RNA sequencing (RNA-seq) and proteomics, are often applied to resolve the etiology of complex diseases, and to develop diagnostics and treatments. Singularly, these avenues often fail to reflect the true spectrum of disease, but combined, the picture becomes clearer. To this end, there is a strong interest in the genomics community to understand transcriptional regulation, including alternative splicing, to improve our understanding of the underlying regulatory mechanisms of disease. Alternative splicing events affect nearly 85% of all protein-coding genes in the human genome (Page et al., 2025), and aberrant splicing events have been associated with various disease states, including cancer and neurodegenerative disease (Wen et al., 2015; Nikom and Zheng, 2023). This connection indicates the importance of characterizing the diversity of RNA isoforms, hereby referred to as the “isonome”, and determining their role across human health and disease.

Long-read sequencing technologies, such as Oxford Nanopore Technologies (ONT), enable researchers to detect both gene and isoform expression patterns as longer reads can more accurately measure full-length transcripts (Tilgner et al., 2014). This is an improvement over standard short-read technologies, which struggle to measure isoform-level expression (Steijger et al., 2013). Long-read sequencing has shown great promise and was named the Method of the Year by Nature Methods in 2023 (Marx, 2023) due to its improved genome assembly in repetitive regions, such as those involved in gene regulation. Several labs recently used bulk long-read sequencing to discover thousands of new RNA isoforms (Glinos et al., 2022; Leung et al., 2021; Heberle et al., 2025), among other findings, including our own work in a small cohort of Alzheimer’s disease (AD) cases and controls, where we identified 53 novel isoforms from disease-associated genes and demonstrated the importance of performing differential expression at the isoform level, not just the gene level (Heberle et al., 2025).

While bulk RNA sequencing has great value, being able to characterize and quantify expression within individual cells via single-cell RNA-seq (scRNA-seq), provides crucial context into cell-specific mechanisms. Researchers have used short-read scRNA-seq for many years, and it has become a powerful tool for disease research, enabling gene-level analyses at single-cell resolution. These studies offer direct insight into downstream cellular mechanisms for specific, critical cell populations unidentifiable via bulk approaches.

scRNA-seq typically uses short-read sequencing, however, which collapses expression measurements across all RNA isoforms for a given gene into a single gene expression measurement, ignoring isoform differences that may provide critical insight into molecular dysfunction (Page et al., 2025; Heberle et al., 2025). Combining scRNA-seq and long-read RNA-seq approaches allows a precise cellular study of isoform expression, offering unprecedented clarity into genomics, disease mechanisms, and potential drug targets. The integration of single-cell and long-read technologies has been performed in a relatively few number of studies (Shiau et al., 2023; Kumari et al., 2024; Yang et al., 2023; Lebrigand et al., 2023; Joglekar et al., 2024; Byrne et al., 2024; Hardwick et al., 2022; Volden et al., 2018; Volden and Vollmers, 2022; Shi et al., 2023; Boileau et al., 2022; Joglekar et al., 2021; Hazzard et al., 2022; Thijssen et al., 2022; Singh et al., 2019; Tian et al., 2021; Rebboah et al., 2021; Philpott et al., 2023). The 10X Genomics droplet-based system is the most established and common among single-cell long-read studies (Yang et al., 2023; Lebrigand et al., 2023; Joglekar et al., 2024; Byrne et al., 2024; Hardwick et al., 2022; Volden et al., 2018; Volden and Vollmers, 2022; Shi et al., 2023; Boileau et al., 2022; Joglekar et al., 2021; Hazzard et al., 2022; Thijssen et al., 2022; Singh et al., 2019), but benchtop-friendly approaches (Rebboah et al., 2021; Hashimshony et al., 2016; Muraro et al., 2016; Clark et al., 2023; Hashimshony et al., 2012) make single-cell approaches more accessible, deviating from the expensive microfluidic machinery that is prone to clogging by cell clumps or debris and reducing the cost per sample. To address this, an array of new benchtop-friendly microfluidic-free approaches to scRNA-seq have recently become available, including those from Fluent Biosciences (recently acquired by Illumina) (Clark et al., 2023), Parse Biosciences (Rebboah et al., 2021), Universal Sequencing (Garcia-Bassets et al., 2024), csGenomics (Marafini et al., 2024), and Scale Biosciences (recently acquired by 10x Genomics).

Although long reads have many advantages, short reads are often used to supplement long reads in single-cell sequencing because the short reads: (1) correct sequencing errors in the long reads (the higher error rate in long-read sequencing makes confidently identifying barcodes challenging); and (2) help identify cluster cell-types with the increased depth. The read depth can be a challenge with long-read single-cell approaches due to cost. Read depth from long reads is not necessarily insufficient when analyzing data at the gene level (i.e., collapsing all isoforms into a single expression measurement), but it becomes a challenge at the isoform level. Thus, the same number of reads is getting split across a dramatically higher number of dimensions (i.e., expression values). For example, a recent study by Page et al. (2025), using data from Glinos et al. (2022), demonstrated that 13,236 unique genes are regularly expressed in the cerebellum, resulting in 22,522 uniquely expressed isoforms. Indeed, to truly grasp biology at both the single-cell and isoform level will require extremely deep long-read sequencing.

Here, we present: (1) a single-cell protocol that has been adapted for use with ONT long reads, (2) a bespoke computational approach (see Data and code availability) to identify barcodes and maximize read recovery in long-read single-cell approaches using ONT, and (3) proof-of-principle results demonstrating the value of this approach using data that are biologically relevant to peripheral blood mononuclear cells (PBMCs). Specifically, we adapted the V4.0PLUS PIPseq protocol originally developed for short-read sequencing by Fluent Biosciences (Clark et al., 2023) to be compatible with long-read nanopore sequencing, combining the strengths of single-cell approaches with long-read sequencing in a benchtop-friendly design; this required small but essential modifications to the protocol and our custom computational approach to identify barcodes and maximize read recovery that is conceptually simple but computationally challenging. In doing so, we enable isoform expression analysis with cell-type specificity. For this study, we chose to use PBMCs to validate our novel adaptation of long-read PIPseq, given their common use as a clinical sample, cellular diversity, ease of collection, abundance, and the absence of well-powered studies on the single-cell long-read level.

## Results

### Adapted PIPseq protocol enables bench-top long-read single-cell sequencing

**FIGURE 1:**
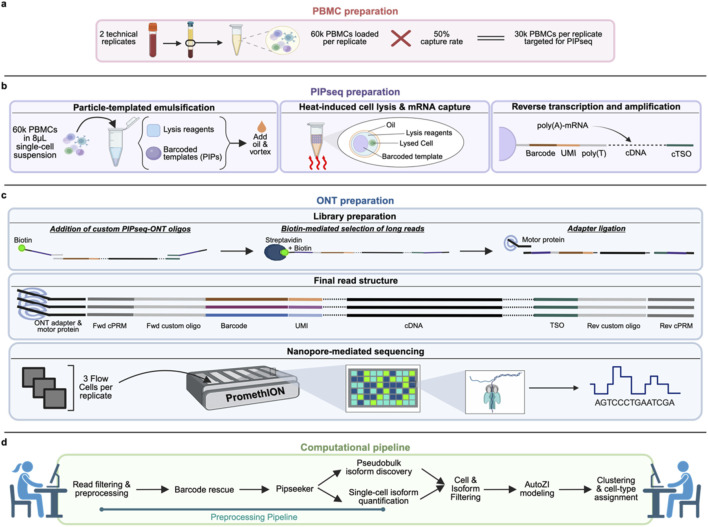
Adapted PIPseq protocol enables bench-top long-read single-cell sequencing. **(a)** Buffy coat from whole blood was processed and 30k cells per replicate were targeted for capture. **(b)** Single cells were encapsulated in lipid droplets with barcoded templates, lysed, and mRNA was captured for reverse transcription. Resulting barcoded cDNA preserved cell-of-origin information during subsequent whole-transcriptome amplification. **(c)** Barcoded cDNA underwent quality assurance, adapter addition, biotin-mediated enrichment of long fragments, and preparation for PromethION sequencing. **(d)** Reads underwent quality filtering, barcodes were rescued, and reads were processed with PIPseeker and fed into pipelines for bulk-level isoform discovery and single-cell isoform quantification. Clustering and cell-type analyses were then performed. *Figure created at* BioRender.com.

In this study, we adapted the PIPseq (Clark et al., 2023) T20 3′ Single Cell RNA Kit developed by Fluent Biosciences for long-read sequencing using ONT-based sequencing (Figure 1; kit now marketed as Illumina Single Cell 3′ RNA Prep, with updated chemistry) and performed sequencing on two PBMC technical replicates (three PromethION flow cells per replicate) across one of the largest numbers of cells in a single-cell long-read study to date. We loaded 60,000 PBMCs per technical replicate to target 30,000 PBMCs (50% capture rate; Figure 1a), the upper limit of the PIPseq T20 chemistry. The PBMCs were isolated from whole blood immediately after collection to preserve RNA integrity and minimize transcriptomic changes. We systematically modified the PIPseq protocol through several key steps to maintain mRNA and cDNA read length (Figures 1b,c) and ensure compatibility with downstream ONT sequencing. Complete details are included in the Methods, but briefly, this was done through a series of key modifications: (1) where possible, we replaced standard pipette tips with wide-bore tips to reduce shear forces while handling fluids; and (2) we eliminated vortexing steps where possible, opting for gentler mixing methods including tube inversion, flicking, or careful pipetting. These steps were especially utilized after barcoded cDNA was removed from the protective PIP environment. To bridge cDNA produced from the modified PIPseq protocol with an improved ONT single-cell protocol, we introduced custom oligos designed for compatibility with both via overlapping sequences primed with the PIPseq Whole Transcriptome Amplification (WTA) Primer to the ONT-specific cDNA primer (cPRM). Detailed sequences can be found in the “Custom oligos for adaptation of PIPseq cDNA for ONT sequencing” section. The samples were then prepared for nanopore sequencing using the PCR-cDNA Sequencing Kit V14 with a modified single-cell preparation optimized for preserving read length, as described in Methods (Figure 1c).

Following sequencing, we processed the reads for quality control, demultiplexed reads into individual cells, and created expression matrices (Figure 1d). Briefly, we performed the following preprocessing steps: (1) filtered out low-quality reads (quality score <9) and oriented and rescued full-length reads with *pychopper*; (2) corrected and rescued barcodes using a modified PIPseeker workflow optimized for long-reads; (3) rescued reads with imperfect barcodes from sequencing errors; and (4) generated pseudo paired-end reads for filtering and demultiplexing using PIPseeker.

### Bulk-level characterization reveals isoform expression landscape in PBMCs

Before performing single-cell analyses, we first characterized the gene and isoform expression landscape in PBMCs at the bulk level to better understand the nature of the peripheral immune cells, including which isoforms from key PBMC genes are driving their underlying biology. Our initial analyses characterized RNA isoform expression characteristics; this included performing isoform discovery using Bambu (Dyer et al., 2025) across all cells for both samples to maximize evidence of new isoforms.

Sequencing generated a total of 275.8M and 216M raw reads for each respective sample (Table 1), yielding the largest and most extensive long-read PBMC dataset by number of cells from a single donor to date (Volden and Vollmers, 2022; Inamo et al., 2024). After quality filtering and alignment, approximately 121.8M and 80.0M reads remained, enabling high-confidence isoform detection. The ∼36% loss in reads from those with a passing quality score to those present after trimming, processing, and barcode rescuing is largely due to the discarding reads with ambiguous and unidentifiable barcodes (Table 1; Supplementary Table S1) The mean read length N50s were 658.7 and 664.7, respectively, while the median read lengths were 476.7 and 501.3, respectively.

**TABLE 1:** Read metrics from PBMC samples collected across 3 flow cells per sample. Values signify metrics from ONT sequencing, filtering, and alignment aggregated across the three flow cells used to sequence each PBMC replicate. Full metrics after each step can be found in Supplementary Table S1.

​	PBMC1	PBMC2
N reads (total)	275,827,438	215,987,216
N reads with quality score >9 (ONT)	228,920,664	163,153,766
N trimmed and processed passed reads	153,038,291	99,777,656
N reads aligned (MAPQ>10)	121,867,484	80,033,261
N50 FASTQ (mean across flow cells, nt)	658.7	664.7
Median read length (nt)	476.7	501.3

Abbreviations: ONT, Oxford Nanopore Technologies; MAPQ, mapping quality score; nt, nucleotides.

We identified 126 new isoforms, where 59 were from previously annotated gene bodies (new from known), and 67 were from previously unidentified gene bodies (new from new). Genes were identified as “new” when Bambu identified transcript structures, with consistent expression, at loci that were not associated with known Ensembl (Dyer et al., 2025) Gene IDs. Similarly, new isoforms were defined as those with unique splice junction patterns distinct from annotated Ensembl Transcript IDs in the given annotation (v113). Of these 126 isoforms, 16 were independently identified in prior long-read studies using different sample types (Glinos et al., 2022; Leung et al., 2021; Heberle et al., 2025) (Supplementary Figure S1), supporting the validity of these transcripts. Seven of the novel transcripts were predicted to be protein-coding, according to analyses using the Coding Potential Calculator 2 (Kang et al., 2017; Kong et al., 2007; Zong et al., 2016) (Supplementary File S1).

Of the 58 known genes with a new isoform, 26 were RNA genes and two were pseudogenes, while the remaining 30 were protein-coding genes. Among these, two of the RNA genes (*TALAM1*, *ADD3-AS1*), though noncoding, have reported regulatory roles in gene expression and cellular signaling, sometimes affecting their neighboring genes, such as in the case of *TALAM1* (Zong et al., 2016; Ye et al., 2022). According to GeneCaRNA (Aggarwal et al., 2024; Barshir et al., 2021) (v5.25), five of the RNA genes are known antisense transcripts, a role that is predicted to regulate the sense gene (Najafi et al., 2022; Pelechano and Steinmetz, 2013). Similarly, two are known sense intronic transcripts, which originate within a gene’s intron and can be precursors for small non-coding RNAs or can regulate its host gene (St Laurent et al., 2012; Knauss and Sun, 2013). These observations highlight the utility of RNA genes often ignored in genomics research, which require further study to truly understand their role.

**FIGURE 2:**
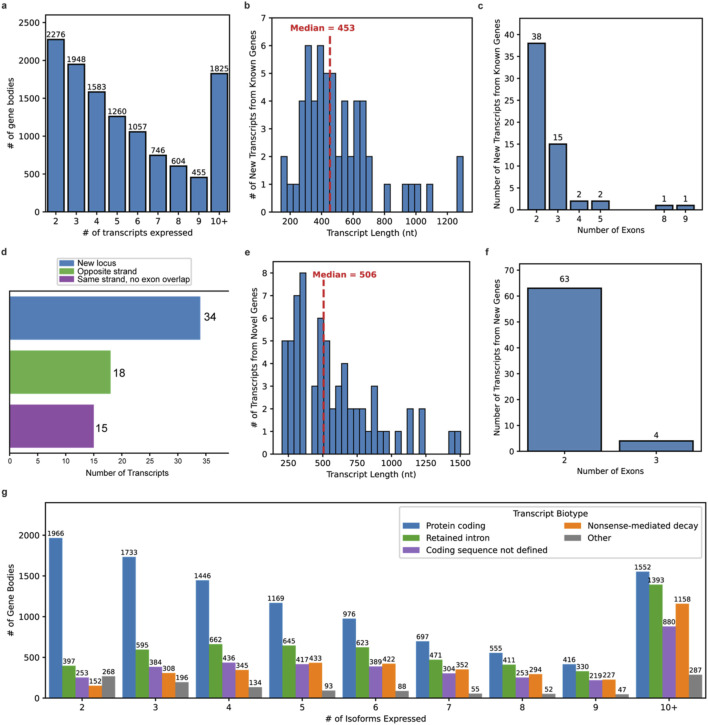
Bulk-level characterization of transcripts from long-read single-cell PBMC data after QC. **(a)** Number of gene bodies expressing more than one isoform. **(b)** Transcript length distribution (nt) for new isoforms found in known genes (median = 453 nt, range = 142–1300). **(c)** Exon number distribution for new isoforms from known genes. **(d)** Bar plot demonstrating genomic position of new transcripts in relation to known genes and transcripts. **(e)** Transcript length distribution for new isoforms from new genes (median = 506 nt, range = 204–1504). **(f)** Exon number distribution for new isoforms from new genes. **(g)** Bar plot of gene bodies by isoform number, as in 2a, subdivided by transcript biotype. The “Other” Category includes biotypes such as long non-coding RNAs and pseudogenes.

Of the genes that expressed multiple isoforms, only a small proportion, 19.3%, expressed only two isoforms, however 50.6% expressed ≥5 isoforms and 15.5% expressed ≥10 isoforms (Figure 2a). New transcripts from known genes were relatively short, with a median of 453 nucleotides (Figure 2b), compared to the overall median length for all isoforms in our dataset, which was 1737 nt across both samples (Supplementary Figure S2). Although all the new isoforms from known genes exhibited multiple exons in the novel transcript, ∼64% of these transcripts contained just two exons (Figure 2c).

We also investigated the genomic position of new isoforms from new gene bodies and found that 26.9% of the new isoforms resided on the opposite strand of the known gene, while 22.4% resided on the same strand, but with no exonic overlap (Figure 2d). The remaining 50.7% were identified as entirely new loci, which could be important for better understanding peripheral immune cells. These new transcripts from previously unannotated genes had a median transcript length of 506 nt (Figure 2e). In these new isoforms from new gene bodies, ∼94% contained just two loci (Figure 2f) consistent with the trend that we identified in our novel isoforms from known genes (Figure 2C). Given the relatively short length of many of our new isoforms and our short median read length (Table 1), it is possible some may be incomplete fragments of a larger novel isoform. It is unlikely, however, that our novel isoforms are fragments of known isoforms since Bambu’s novel isoform detection relies on different splice junctions rather than alternative start or stop sites to account for incomplete transcripts (Chen et al., 2023).

We identified that most of the transcripts expressed in the PBMCs were annotated as protein-coding, according to Ensembl (HG38 release 113) (Dyer et al., 2025) regardless of the number of isoforms expressed in the gene body (Figure 2g). However, as the number of isoforms expressed within a single gene increases across the distribution, we noticed that fewer protein-coding isoforms are expressed in favor of a higher proportion of other transcript biotypes, such as those with retained introns or annotated as nonsense-mediated decay. Several transcripts also appear to have undefined coding sequences (e.g., *CD33*, *AHR*), reflecting a remaining gap in our understanding of functional isoform expression.

### Canonical marker genes exhibit high isoform diversity in PBMC single-cell data

**FIGURE 3:**
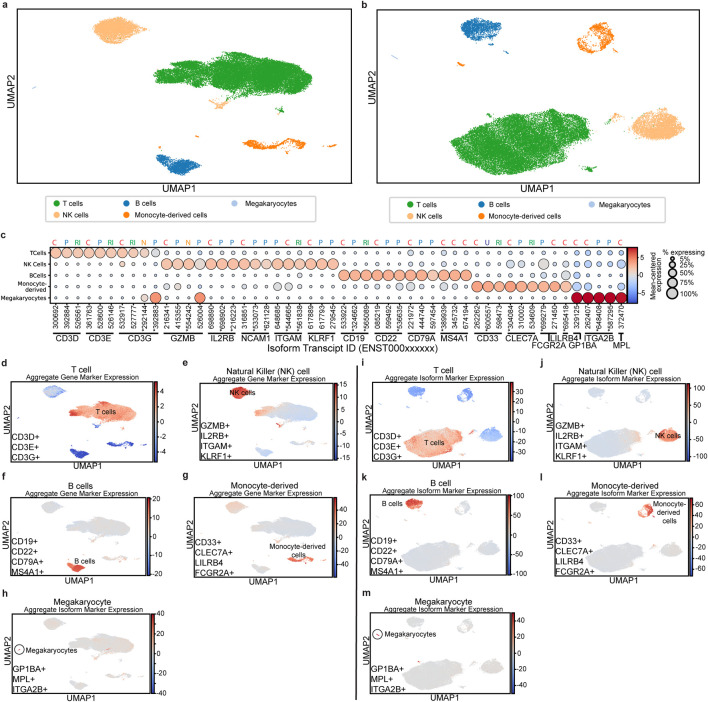
Immune cell clustering and marker-based cell-type assignment at the gene- and isoform-level. **(a,b)** UMAP projections of cell clustering, colored by assigned cell-type: **(a)** gene-level clustering and **(b)** isoform-level clustering. **(c)** Dot plot of up to three representative isoforms per gene, selected as the most highly expressed isoforms by raw counts (Supplementary File S2), and including any isoforms enriched in a non-canonical cell-type (e.g., *GZMB* expression in megakaryocytes). The x-axis represents isoform transcript ID (ENST00000xxxxxx), with the last 6 digits of the isoform ID printed to differentiate isoforms and their respective dot. See Supplementary Figure S3 for all isoforms expressed. Genes marked with an asterisk (*) indicate isoforms not recovered in unique-count analysis alone, which may reflect read counts split between multiple similar isoforms or insufficient full-length reads to resolve isoform ambiguity. Circle size indicates the percentage of cells in each cluster expressing the marker, and circle color indicates fold change from average expression. Transcript type is marked above each transcript (C, Canonical Protein; P, Alternative Protein; N, nonsense-mediated decay; RI, Retained intron; noncoding; U, Unknown coding status). **(d–h)** Gene-level UMAPs of aggregate marker expression for **(d)** T cells (*CD3D*, *CD3E*, *CD3G*); **(e)** Natural Killer (NK) cells (*GZMB*, *IL2RB*, ITGAM, *KLRF1*); **(f)** B cells (*CD19*, *CD22*, *CD79A*, *MS4A1*); **(g)** Monocyte-derived cells (*CD33*, *CLEC7A*, *FCGR2A*, *LILRB4*); and **(h)** Megakaryocytes (*GP1BA*, *ITGA2B*, *MPL*). **(i–m)** Isoform-level UMAPs of aggregate marker expression for **(i)** T cells, **(j)** NK cells, **(k)** B cells, **(l)** Monocyte-derived cells, and **(m)** Megakaryocytes.

After performing isoform discovery in the bulk-level analysis, we proceeded to perform the single-cell analyses, where we performed rigorous cell and isoform filtering, ultimately resulting in of 11,352 and 21,666 cells for PBMC1 and PBMC2, respectively. We utilized AutoZI to adjust for zero inflation, which occurs when many genes or isoforms exhibit zero counts more often than expected due to limited read depth or capture. This phenomenon is more common in single-cell datasets since reads are split across individual cells rather than pooled (i.e. bulk-level data) making it difficult to directly measure expression consistently across the cells. Unlike other methods, AutoZI identifies genes susceptible to zero inflation (Supplementary Table S3) and applies a zero-inflated negative binomial (ZINB) (Greene, 1994) distribution to estimate the true expression values for individual genes and isoforms (Clivio et al., 2019). After zero-inflation correction, we constructed a K-nearest neighbor (KNN) graph with 20 neighbors, performed unsupervised clustering with the Leiden algorithm (resolution: 0.06), and visualized clusters using UMAP. Using known markers (Supplementary Table S4 and Methods), we identified five clusters with major immune cell types at both the gene (Figure 3a) and isoform levels (Figure 3b); in order of abundance, these are T cells (24,757 cells; 74.98%), natural killer (NK) cells (4,623; 14.00%), B cells (2,231; 6.76%), monocyte-derived cells (1,339; 4.06%), and megakaryocytes (68; 0.21%). While canonically considered rare for megakaryocytes to be found in the circulation, except in the instance of bone marrow-related diseases, we identified a very small population through genetic markers (*GP1BA*, *ITGA2B*, and *MPL*) distinct from circulating neutrophils; our findings concur with previous scRNA-seq work that observed megakaryocytes in circulation accounting for 0.3%–0.4% of total PBMCs (Zheng et al., 2017; Zhang et al., 2022).

We then assessed isoform expression differences by cell-type across marker genes. To understand biology at the isoform level, we first characterized the landscape by defining cell types at the RNA isoform level (i.e., marker isoforms) rather than solely at the gene level (i.e., marker genes). The ultimate goal is to eventually determine which specific protein isoforms drive cellular biology for each cell type and state; this will have important implications for molecular studies (e.g., those that rely on antibodies). Due to the challenges with per-cell depth associated with long-read sequencing, these analyses are intended to identify trend-level isoform usage patterns, rather than definitive differential expression, and should be interpreted conservatively.

As expected, marker genes and their individual isoforms were either enriched in or specific to their respective cell types (Figure 3c), but we were surprised by the number of unique isoforms expressed by each marker gene. Of the 19 primary cell-type marker genes used in this study, 15 exhibited multiple detectable unique isoforms, where half expressed ≥5 isoforms and three expressed ≥10 (Supplementary Figures S3, S6; Supplementary File S1). Additionally, two cell-type-specific marker genes (*CD3G* and *GZMB*) exhibited potential isoform enrichment in megakaryocytes (Figure 3c). Both genes demonstrated higher relative abundance of an alternative protein-coding isoform (*CD3G*: ENST00000392883; *GZMB*: ENST000005260004) in megakaryocytes, compared to other cell types.

We also generated aggregate “gene scores” for each set of marker genes, which clearly indicate each cluster (Figures 3d–h). Specifically, the aggregate gene scores are generated by combining expression values for each set of marker genes into a single expression measurement to maximize cluster contrast (e.g., *CD3G*, *CD3D*, and *CD3E* for T cells). Similarly, we aggregated all marker isoforms into “isoform scores” to assign a cluster to their respective cell identity (Figures 3i–m) (Young and Behjati, 2020).

### Cell-type-specific isoforms of *CD3G* and *GZMB* suggest functional divergence or loss of function of non-canonical proteins from canonical functions

As proof of principle for the importance of understanding isoform differences between cell types, we compared the protein sequences for the *CD3G* and GZMB isoforms detected in megakaryocytes to their canonical forms. For consistency and clarity, we adhere to Ensembl’s definition of a canonical transcript, as described in their documentation (Ensembl, 2025). Briefly, a canonical transcript is designed as a single representative transcript per locus, based on evidence including expression, coding sequence features, transcript length, and agreement with external annotation resources (e.g., NCBI, UniProt). If our detected isoform enrichment is authentic to the biology, the differences between proteins suggest potential functional divergence or loss of function.

**FIGURE 4:**
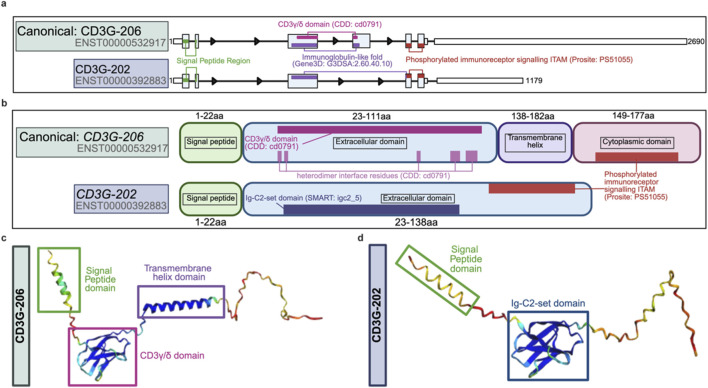
Protein-coding region significance and predicted structures of CD3G isoforms. **(a)** CD3G-206 and CD3G-202 RNA isoform structures, encoding domains including the signal peptide, immunoglobulin-like fold, and immunoreceptor tyrosine-based active motif (ITAM) site. Loss of exon 4 in CD3G-202 changes immunoglobulin-like specificity, replacing the CD3γ/δ domain with a less specific immunoglobulin-like fold. **(b)** Domain architecture of CD3G-206 and CD3G-202 proteins, including heterodimer interface residues (extracellular domain), transmembrane helix, and ITAM (cytoplasmic domain). Loss of the transmembrane helix region exhibited in CD3G-202 alters ITAM region localization relative to the membrane. **(c,d)** Predicted protein structure of CD3G-206 **(c)** and CD3G-202, missing the transmembrane helix domain **(d)**, modeled by AlphaFold using the protein sequences in Supplementary Table S6, with confidence scores colored from blue (high) to red (low) and highlighting isoform-specific changes in protein folding. AlphaFold output files including.PDB of the final structure can be found on Zenodo (Ebbert et al., 2025), as described in Data and Code Availability. Created at BioRender.com.

*CD3G* is a canonical marker for T cells that encodes the gamma subunit of the CD3 protein complex, a crucial receptor in T cell receptor signaling (Oelen et al., 2022). As expected, the canonical isoform (CD3G-206, ENST00000532917) is enriched in T cells, but an alternative protein-coding isoform (CD3G-202, ENST00000392883) may be enriched in megakaryocytes. InterProScan (v5.75-106.0) (Jones et al., 2014; Blum et al., 2025) and Sherbrooke Alternative Protein Feature IdentificatoR (SAPFIR) (Zhou et al., 2022) analyses suggested that the canonical isoform (CD3G-206) contains a signal peptide region, an extracellular IgC1 domain (Conserved Domain Database (Wang et al., 2023) (CDD): IgC1_CD3_gamma_delta), a transmembrane helix domain, and a cytoplasmic tail containing an immunoreceptor tyrosine-based activation (ITAM) motif (Figures 4a,b). Based on InterProScan and CDD results, the protein sequence produced from the CD3G-202 isoform matches a sequence motif associated with a generic immunoglobulin-like fold, appearing to be missing the heterodimer interface (CDD: cd07691) and Ig strands A, C′, and D (Figure 4b). CD3G-202 is missing the transmembrane domain, and the ITAM motif is in the extracellular domain; these features combined with the signal peptide region, a region responsible for localization to the endoplasmic reticulum for packaging into the secretory system, indicates this may be secreted rather than a membrane-bound form of the protein. These predictions were supported by results from DeepTMHMM (v1.0.44) (Hallgren et al., 2022), which predicted a transmembrane helix in CD3G-206 (aa 118-136), but none in CD3G-202. The physical structure predicted by AlphaFold2 appears to support these conclusions, based on a well-defined structure in CD3G-206 (Figure 4c), and the absence of a transmembrane helix in the CD3G-202 structure (Figure 4d). Why CD3G-202 may be expressed in megakaryocytes, or why it is ever expressed, along with any potential function, is unknown. This is a clear example of the need to understand biology at the next level of isoforms, and highlights the hypothesis-generating potential of our framework.

Secreted alternative proteins without canonical functionality have been identified in the transmembrane receptor RAGE (Maillard-Lefebvre et al., 2009; Ciccocioppo et al., 2015) and receptor kinase VEGFR1 (Sarabipour et al., 2025), which act as dominant-negative decoys to bind substrates. Truncated non-receptor protein-coding isoforms that lose their canonical functional domains have also been reported as immune regulators; for example, the STING (TMEM173) protein produces isoforms lacking an N-terminal transmembrane domain, disrupting proper cellular localization and negatively regulating canonical STING signaling (Deng J. et al., 2025). Thus, potential functions of CD3G-202-derived proteins could be (1) to compete with canonical CD3G-206 in the secretory machinery, potentially slowing the protein’s release from T cells; (2) to act as a decoy to bind substrates or target sites competitively, modulating activity in extracellular sites; or (3) nonfunctional.

We next examined *GZMB*, which may also have preferentially expressed a specific isoform in megakaryocytes. *GZMB* encodes the Granzyme B serine protease, a cytotoxic secretory molecule associated with inducing apoptosis in other cells (Trapani and Sutton, 2003), which acts as a marker gene for NK cells and cytotoxic T cell subsets. As expected, the canonical isoform, GZMB-201 (ENST00000216341) is enriched in NK cells, whereas GZMB-204 (ENST000005260004) is potentially enriched in megakaryocytes. These two isoforms are identical through the 69th amino acid (aa), part of exon 3, after which point GZMB-204 diverges, resulting in a much shorter protein sequence (GZMB-201: 247aa vs. GZMB-204: 90aa). Structural studies into the catalytic mechanism underlying the Granzyme B function have determined the utilization of a catalytic triad (His59, Asp103, Ser198) (Tripathi et al., 2022), all of which are required for canonical activation of the protease. Both isoforms appear to contain the trypsin histidine active site encoded in exon 2 (Supplementary Figure S4a, b). GZMB-204, however, lacks exons 4 and 5 (Supplementary Figures S4a, b), which encode for the trypsin serine active site (197aa-208aa), a domain functionally responsible for targeting the peptide bond of a target protein (Tripathi et al., 2022). Although not highlighted by InterProScan, the truncated GZMB-204 also lacks the Asp103 active site, which is critical for stabilization of the protonation state (Tripathi et al., 2022). Because GZMB-204 is missing Asp103 and Ser198, the catalytic triad would likely not be formed, suggesting reduced or absent protease activity. AlphaFold2-based predictions (Jumper et al., 2021; Mirdita et al., 2022) also suggest major differences in the protein structure, with two β-barrels in GZMB-201 (Supplementary Figure S4c), and only one in GZMB-204 (Supplementary Figure S4d). As the juncture of these two β-barrels is where substrates are bound, we expect this change would significantly impact fuction. However, with an intact signal peptide, the GZMB-204 protein could still be secreted, possibly with an impaired protease function relative to its canonical form.

Additionally, NK cells showed enrichment for several *GZMB* isoforms with differing structures, including GZMB-203 (ENST00000415355) (Figure 3c) and GZMB-202 (ENST00000382540) (Supplementary Figure S3). GZMB-203 retains a functional domain identical to that of the canonical isoform, but is missing a signal peptide. Therefore, this form of *GZMB* would be unlikely to be secreted and may be degraded by the proteasome. NK cells also express GZMB-202, an isoform missing exon 3, which contains Asp103, essential to stabilize and activate the catalytic triad, leaving the protein likely to be catalytically inactive. While still likely secreted, GZMB-204 and -202 could contribute to the extracellular granzyme pool, limiting tissue damage without contributing to cytotoxic activity. As postulated for the secreted form of *CD3G,* GZMB-204 and -202 may function as decoy isoforms or as immune regulators, biological mechanisms that have been proposed for alternative isoforms from other genes as potential biomarkers and treatments in diseases such as SARS-CoV-2 infection (Havranek et al., 2023).

Because *GZMB* and *CD3G* are canonical markers of NK and T cells, respectively, we evaluated whether substantial ambient RNA contamination in our dataset could explain the enrichment of these markers in megakaryocytes. Using SoupX (Young and Behjati, 2020), we determined that the global contamination was estimated to be ∼1.1%, and was consistent across all cell types (Supplementary Table S5, median percent contamination range: 0.9%–1.1%). We also compared the total counts per cell from our corrected and uncorrected count matrices, and found minimal differences in total counts per cell (median difference = 20.5 reads; Supplementary Table S5). Negative minimum values reflect per-cell over-correction during SoupX modeling, where contamination count estimations exceed the raw counts of a given isoform within a single cell.

After SoupX modeling correction, *CD3G* signal remained detectable in megakaryocytes, though at lower levels than in T cells. Even overall *CD3G* expression in T cell populations exhibited low counts (31.4%–42.08% of cells expressing), reflecting the depth limitations of long-read single-cell sequencing. However, relative to non-T cells, *CD3G* remained strongly enriched and SoupX changed the expression profile by less than 1%, indicating a robust primary enrichment pattern. In contrast, *GZMB* signal was at or below the detection threshold in the raw matrix and became ‘expressed’ only after applying AutoZI denoising, our approach applied to address zero-inflation due to limited sequencing depth. Because AutoZI modeling borrows transcriptional patterns across similar cells, the *GZMB* signal can reflect model-based inference rather than direct detection and should be interpreted cautiously.

These findings indicate that a small amount of ambient RNA contamination is present, as is expected in droplet-based approaches, and given the limited cell number, sequencing depth, and reliance on denoising for *GZMB* detection, these observations should be considered hypothesis-generating. The *CD3G* enrichment pattern, however, is unlikely to be fully explained by ambient RNA contamination alone. Whether the individual isoforms for each marker gene are performing a unique function (even if subtle) is not yet known, but understanding when, where, and why individual isoforms are expressed will be essential to better understanding gene function and cellular behavior.

These examples underscore the importance of isoform-level analyses and are critical for understanding endogenous modulation of immune responses in humans. We also highlight the utility of the above-described tools for forming specific hypotheses that predict differences in functionality between isoforms from the same gene. Even independent of the specific cell-type context, the structural differences between these isoforms within the same gene highlight that gene-level annotation is insufficient to infer protein function.

### T cell subtype characterization demonstrates subtype-dependent alternative splicing and isoform usage

With the added resolution provided by the large number of cells and the use of long reads, we next focused on subtype clustering for T cells. T cells are the most abundant cellular population in our dataset and are known to be biologically heterogeneous, exhibiting diverse roles in the adaptive immune system (Chaplin, , 2010), making them a logical choice to use to evaluate the heterogeneity in our data as a proof-of-principle.

**FIGURE 5:**
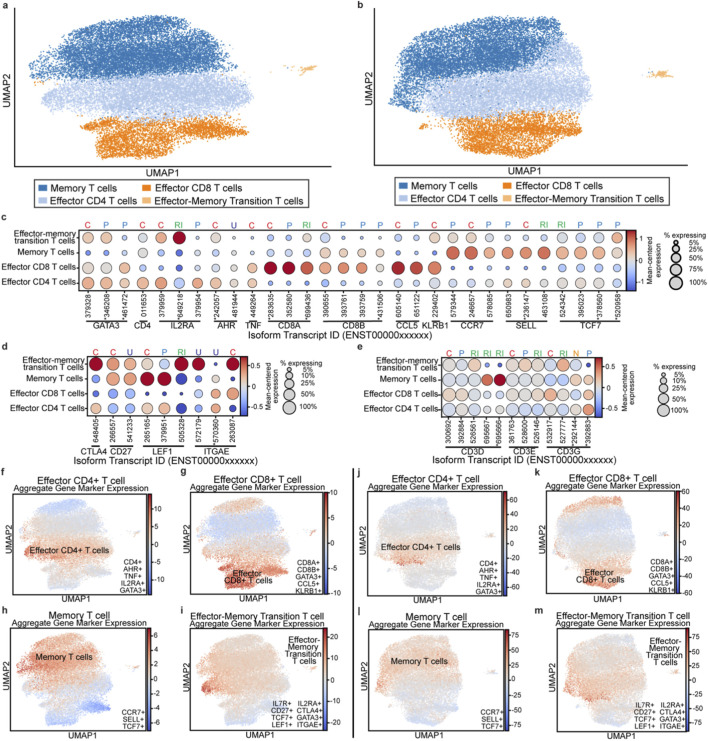
T cell subtype clustering by canonical markers at the gene- and isoform-level. **(a)** Gene-level UMAP projection of T cell subtype clusters. **(b)** Isoform-level UMAP projection of T cell subtype clusters. **(c–e)** Dot plots of up to three representative isoforms per gene, selected as the most highly expressed isoforms by raw counts (Supplementary Figure S7), and including any isoforms enriched in a non-canonical cell-type. X axis represents isoform transcript ID (ENST00000xxxxxx) with the last 6 digits of the isoform ID printed to differentiate isoforms and their respective dot. See Supplementary Figure S7 for all isoforms expressed. Colored boxes correspond to cluster-defining markers. Genes marked with an asterisk (*) indicate isoforms not recovered in unique-count analysis alone, which may reflect read counts split between multiple similar isoforms or insufficient full-length reads to resolve isoform ambiguity. Circle size indicates the percentage of cells in each cluster expressing the marker; circle color indicates fold change relative to average expression. Transcript type is indicated above each transcript (C, Canonical Protein; P, Alternative Protein; N, nonsense-mediated decay; RI, Retained intron, noncoding; U, Unknown coding sequence). Plots are split up by **(c)** Main T cell sub-type markers (Memory T cells, Effector CD4 and CD8 T cells), **(d)** markers specific to effector-memory transition T cells, and **(e)** general T cell markers. **(f–i)** Gene-level expression aggregation on the single-cell level in **(f)** CD4^+^ effector T cells, **(g)** cytotoxic/effector CD8^+^ T cells, **(h)** memory T cells, and **(i)** effector-memory transition T cells. **(j–m)** Isoform-level expression aggregation on the single-cell level in **(j)** CD4^+^ effector T cells, **(k)** cytotoxic/CD8^+^ effector T cells, **(l)** memory T cells, and **(m)** effector-memory transition T cells.

We analyzed the previously identified T cell population using a Leiden resolution of 0.26 and identified three major subtypes based on canonical marker genes (Supplementary Table S4; Figures 5a,b): effector CD8^+^ T cells (*CD8A*, *CD8B*, *KLRB1*, *GATA3*, *CCL5*), effector CD4^+^ T cells (*CD4*, *IL2RA*, *TNF*, *AHR*, *GATA3*), and memory T cells (*TCF7*, *CCR7*, *SELL*). The CD8^+^ effector cluster was identified as specifically retaining effector functionality by its expression of known effector and activation genes, *GATA3* (Aggarwal et al., 2024) and *CCL5* (Lu et al., 2022). We identified CD4^+^ and CD8^+^ expression patterns within portions of the memory T cell cluster; however, resolution was insufficient to separate these into sub-groups (Figure 5c). In some genes (*AHR* and *KLRB1)*, one to two isoforms were expressed, though *AHR* has 7 known isoforms. This could be due to a tissue-specific isoform expression not enriched in the blood, as shown for numerous tissues (Glinos et al., 2022). Overall, however, we identified that most of our T cell subtype marker genes expressed multiple isoforms in our dataset, with six expressing at least five isoforms, and two of these genes expressing over 10 isoforms (Supplementary File 2). It is possible that alternative splicing may be dynamically regulated across cell-type or activation state, resulting in preferential isoform expression within a specific context.

*CD8B* isoform usage in our data is an intriguing example of isoform diversity within a single gene, where a non-canonical isoform (CD8B-206, ENST00000431506) encodes a nearly identical protein to that of the canonical isoform (CD8B-203, ENST00000390655), except for a single amino acid change: a phenylalanine (F) in CD8B-203 is replaced by a leucine (L) in CD8B-206 within the cytoplasmic tail region (208aa). Because these two isoforms share most of their sequence, many reads would not be uniquely assignable at the isoform-level, illustrating why unique-only counts can understate true isoform-level differences. Notably, CD8B-206 is more highly expressed in memory T cells than in effector CD8^+^ (cytotoxic) T cells (Figure 5c). Although the protein-coding sequences are nearly identical, these two mRNA isoforms differ in the 3′ untranslated region (3′UTR), where CD8B-206 completely lacks a 3′UTR, according to UTRdb (Lo Giudice et al., 2023) (Supplementary Figure S5). The 3′ UTR region has been shown to regulate mRNA localization, translation, and stability, and exhibits cell-type-specific patterns of 3′UTR length changes upon activation of signaling pathways (Mayr, 2019). 3′UTRs often contain motifs bound by RNA-binding proteins and microRNAs that mediate RNA decay or transcriptional regulation (Geis et al., 2016; Siegel et al., 2022; Koh et al., 2019); therefore, the loss of this region in CD8B-206 may indicate enhanced stability and reduced likelihood of regulation. The presence of a *CD8B* mRNA isoform lacking a 3′UTR in CD8^+^ memory T cells may help sustain expression of this protein in memory T cells, which tend to be long-lived. Consistent with this, prior gene-level studies have shown 3′UTR shortening in proliferating T cells, with CD8^+^ effector memory T cells exhibiting the most pronounced shortening (Qiang et al., 2024). These isoform-specific patterns would not be detectable at the gene level or in bulk long-read sequencing lacking cell-type-resolved resolution, highlighting the unique value of long-read single-cell approaches in providing crucial information on transcriptional patterns in a cell-type-dependent manner.

In addition to these three main clusters, we identified a smaller cluster of cells adjacent to the main T cell population that expressed a mixture of effector/activation markers (*IL2RA, CTLA4, GATA3*) and markers for memory persistence, stem-like, and transition states (*IL7R, CD27, TCF7, and LEF1;* Figure 5d), with isoform specificity. These cells do not express NK-T cell markers (*NCAM1, GZMB*), exhibit unremarkable doublet scores, and show low mitochondrial gene expression (Supplementary Figure S6); these characteristics combined support the validity of a distinct cluster, rather than an artifact. Additionally, the expression of *ITGAE* (CD103; Figure 5d), also enriched in this cluster, is associated with tissue-resident T cells and may indicate that these cells have tissue-resident memory potential. These features are most consistent with a transitional effector-memory T cell cluster.

A distinct cell-type-specific pattern emerged in our effector-memory transition cluster, where marker genes exhibited limited numbers of isoforms compared to other T cell subtypes. Unlike other clusters that typically exhibit high expression of most isoforms of their defining marker genes, this transition cluster highly expressed only 1-2 isoforms per marker gene. For example, while *IL2RA* is highly expressed in effector CD4^+^ T cells among all isoforms, only the canonical isoform (IL2RA-201, ENST00000379959) and an isoform with a retained intron (IL2RA-206, ENST00000649218) were highly expressed in the transition cluster (Figure 5c). Similarly, marker genes of this transition state showed preferential expression of an isoform annotated as retained intron (noncoding) in *LEF1* (LEF1-210; ENST00000505328), instead of the diverse array of isoforms seen in other clusters (Figure 5d). *ITGAE* expression was likewise enriched in this cluster for the canonical form (ITGAE-201; ENST00000263087) and an isoform with an unknown coding sequence (ITGAE-207; ENST00000572179) (Figure 5d). This selective isoform usage may reflect a specialized transcriptional control associated with the transitional state between effector and memory conditions.

Interestingly, we also observed that two large non-coding isoforms of *CD3D* are enriched in memory T cells (Figure 5e). Although the memory subgroup exhibits overlapping expression patterns on the aggregated gene level (Figures 5f–i), our isoform-level analyses (Figures 5j–m) exhibit clear expression differences, highlighting the importance and utility of isoform-level studies to better understand the underlying biology.

### New isoforms exhibit distinct cell-type specificity in expression and structural changes from known isoforms

**FIGURE 6:**
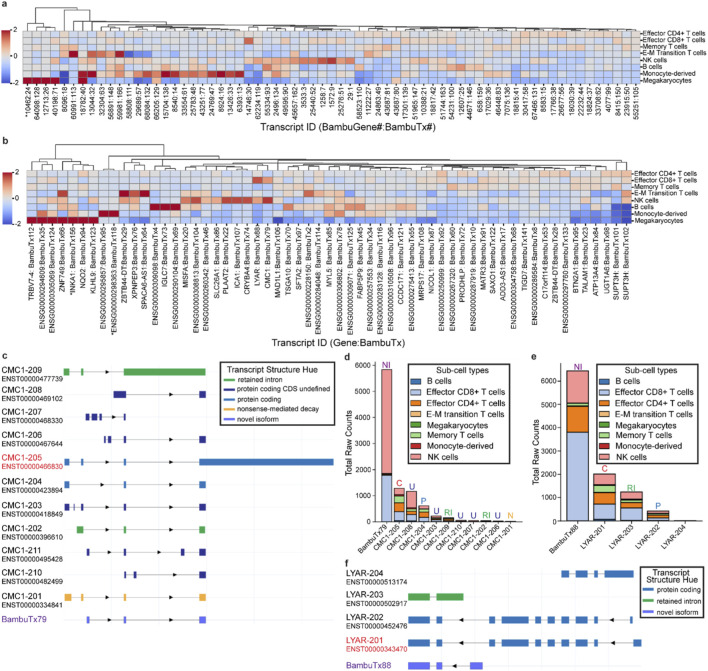
New isoforms from new and known genes exhibit cell-type specific expression patterns and distinct isoform structure. **(a,b)** Heatmaps showing Z-score normalized expression of novel isoforms across major cell-types and T cell sub-types in **(a)** previously unannotated genes and **(b)** known genes. **(c)** Transcript structure models generated with *RNAPysoforms* for *CMC1* isoforms, including the novel transcript BambuTx79 (indicated in purple) and canonical isoform CMC1-205 (indicated by red text). **(d,e)** Raw expression of *CMC1* **(d)** and *LYAR* **(e)** isoforms across major cell-types and T cell sub-types, showing that novel transcripts are the most highly expressed variants in both genes. Transcript biotype is annotated above each isoform including novel isoform (NI), canonical (C), unknown coding sequence (U), protein-coding (P), retained intron (RI), and nonsense-mediated decay (N). **(f)** Transcript structure models for *LYAR* isoforms, including novel BambuTx88.

To identify trends of expression patterns for our newly discovered isoforms from new and known genes, we examined inter-gene clustering for our new isoforms to see which exhibited similar expression patterns (Figures 6a,b). Several of our new genes demonstrated enrichment in specific cell types, with dendrograph clustering appearing fairly distinct for genes enriched in NK cells, megakaryocytes, B cells, monocyte-derived cells, and effector-memory transition T cells (Figure 6a). Although some of these novel genes were enriched in the memory, effector CD4^+^, and effector CD8^+^ T cell clusters, very few exhibited strong, specific enrichment.

Among known genes with novel isoforms,16 expressed only the novel transcript, which may indicate a peripheral blood-specific role. Four of these (*SLC26A1*, *SAXO1*, *SFTA2*, *MISFA*) are not typically expressed in the blood, while two (*CRYBA4*, *IGLC7*) are lowly expressed in the blood according to the Human Protein Atlas (V25.0) (Karlsson et al., 2021). These single-isoform expression patterns may indicate a peripheral blood-specific transcript usage that has been difficult to detect with other sequencing methods. However, several remain unnamed beyond an Ensembl ID or are non-coding RNA genes and are likely to be understudied and therefore not well understood.

Intriguingly, while 40 known genes with new isoforms expressed multiple isoforms, in 26 of these genes, the most highly expressed isoform was our novel one (Supplementary File S2). For two of these genes, *SUPT3H* and *ZBTB44-DT*, two new isoforms were identified, and both exhibited greater overall raw counts than known isoforms (Supplementary File S2). Despite belonging to the same gene, these new isoforms did not exhibit the same expression patterns. Although the new isoforms for *SUPT3H* (BambuTx101 and 102) clustered together on the dendrograph (Figure 6b) and showed consistent enrichment in CD4^+^ and CD8^+^ effector T cells, BambuTx102 was also enriched in NK cells and effector-memory transition T cells, while BambuTx101 was enriched in memory T cells. Additionally, in *ZBTB44-DT*, BambuTx28 showed distinct isoform expression patterns from its sister isoform, BambuTx29, diverging on the dendrograph by several clades and showing enrichment in different cell-types (Figure 6b). BambuTx28 demonstrated enrichment in effector CD4^+^ T cells, while BambuTx29 was enriched in effector-memory Transition T cells and NK cells.

Another gene that exhibits a new transcript as its most highly expressed isoform is *CMC1*, which encodes a highly conserved mitochondrial protein that contributes to the construction of cytochrome c oxidase (complex IV), the last stop in the electron transport chain (Bourens and Barrientos, 2017). Transcript modeling of *CMC1* isoforms using the *RNApysoforms* (Aguzzoli Heberle et al., 2024) package confirmed a novel exon conformation in BambuTx79, showing a structure maintaining an intact exon 2 and 3, but a missing exon 1 and truncated exon 4 (Figure 6c). Interestingly, BambuTx79 also exhibited a distinctive expression pattern from the other isoforms within the gene, displaying enrichment in CD8^+^ T cells and NK cells alone, whereas the other isoforms of *CMC1* have a broader expression pattern across cell-types (Figure 6d). It appears that there is still more to learn about *CMC1*, in part since we notice many transcripts whose protein-coding sequences are unknown (Figure 6d). According to our CPC2 analyses, BambuTx79 is predicted to be noncoding, however, we cannot definitively predict this isoform’s function on this basis alone. Coding-potential predictors are useful first-pass tools, but can miss transcripts encoding proteins with short reading frames or alternative start site (Wright et al., 2022). This transcript exhibits enrichment in cytotoxic cell-types, cells whose activation is associated with increased metabolic activity (Loftus and Finlay, 2016), so a novel isoform in this gene may contribute to regulating this change as a noncoding transcript or could be nonfunctional. Future studies examining ORFs and experimental evidence of translation may provide greater insight into their functionality.

Another gene with a new highly expressed isoform is *LYAR* (Figure 6e), a protein-coding gene with a broad range of roles, including transcriptional regulation, DNA-binding, transcription factor binding activity, and regulation of immune responses (e.g., NFκB) (Yang et al., 2019; Miyazawa et al., 2014). Our new isoform of *LYAR*, BambuTx88, exhibits similar expression patterns to its other isoforms, with most raw counts originating from effector CD8^+^ T cells, with the next largest proportion of reads originating from NK cells, then effector CD4^+^ T cells (Figure 6e). When we modeled the *LYAR* isoform structures, we noticed that the end structure of BambuTx88 matched with exons 9 and 10 seen in LYAR-201 and LYAR-202 but appeared to have an exon 8 that does not align with any other isoform in that location, as the others have a larger intronic region before exon 8, supporting its novelty (Figure 6f). Since the functions of *LYAR* are wide-ranging, this newly identified isoform could have a myriad of different functions that would need to be examined experimentally.

Together, these analyses reveal several previously unannotated isoforms that are not only highly expressed but also have distinct isoform structures from previously annotated isoforms and exhibit defined cell-type-specific expression patterns. Future and deeper studies will be necessary to clarify the functions of the individual isoforms across these important genes.

## Discussion

### Long-read scRNA-seq unlocks new biological insights in human PBMCs

Gene-level scRNA-seq has proven an important tool for understanding transcriptomic profiles in disease and for characterizing heterogeneity across a range of biological processes (Wagner et al., 2016), including immune function (Papalexi and Satija, 2018). However, isoform-level variation within a single gene can vastly impact protein structure and functionality, a detail that is crucial to understanding the underlying biology that has been overlooked because of short-read approaches. Our adapted PIPseq long-read single-cell approach, including our bespoke read recovery pipeline, bridges this gap by directly capturing canonical and non-canonical RNA isoform expression in a cell-type-specific manner, uncovering potential functional variation that cannot be identified via standard gene-level approaches.

In PBMCs, we identified several striking and unexpected trends, including that most of our marker genes simultaneously expressed multiple isoforms in the same cell type (in some cases, over 10), while other marker genes appeared to preferentially express certain isoforms in different cell types. Of the 35 marker genes used in this study, only six expressed just a single isoform (*TNF, KLRB1, CTLA4, MPL, LILRB4, GP1BA),* whereas the rest averaged ∼5.4 isoforms (Supplementary Figures S3, S6, Supplementary File S2). Notably, in megakaryocytes, we identified enrichment for two non-canonical isoforms: (1) an abbreviated *GZMB* isoform missing a domain required for its canonical proteolytic activity, and (2) a secreted form of *CD3G*; both isoforms are overlooked in gene-level data. Additionally, we noticed that for many of our marker genes (*ITGAM, FCGR2A, CCR7, SELL, TCF7*, and *ITGAE*) and genes with newly identified isoforms, the most abundant isoform was not the canonical transcript (Supplementary File S2), underscoring the sometimes-arbitrary nature of canonical definitions and the importance of isoform-level studies to understand the underlying biology. These findings reveal the extensive isoform diversity of immune genes and show how long-read data can provide functional insight into different proteins from a single gene.

Although definitive expression of the *GZMB* and *CD3G* isoforms in our megakaryocyte population would require targeted validation studies, other single-cell studies have characterized heterogeneity underlying megakaryocyte populations (Choudry et al., 2021; Zufferey et al., 2017; Liu et al., 2021; Sun et al., 2021), including up to 14 subtypes (Zhang et al., 2022). Some of these subtypes are tied to immunity and inflammation (Choudry et al., 2021; Zufferey et al., 2017; Liu et al., 2021; Sun et al., 2021), including three reported in a meta-analysis by Zhang et al. (2022) expressing genes associated with T cells, NK cells, major histocompatibility complex class II (MHC-II) signaling, and immune response (*IL32*, *CD52*, *TRAC*). These immune clusters total ∼14% of the proportion of megakaryocytes from PBMCs, so low-level signals from this small sub-population may have been amplified by our AutoZI modeling, explaining the widespread representation. *GZMB* and other immune response genes have also been reportedly expressed in megakaryocytes during immune challenges, such as in sepsis or COVID-19 (Ajanel and Middleton, 2023). These studies may provide some context for why we have identified *GZMB* in our small megakaryocyte population; notably, we are among the first to report a specific isoform potentially enriched from these genes.

While not directly validated here, numerous examples exist demonstrating that different isoforms from the same gene provide valuable insights into human health and disease. For example, isoform-level differences have shown clinical relevance in Alzheimer’s disease, where a tissue-specific isoform of tau, a characteristic pathological marker, outperforms total tau as a predictive blood biomarker of the disease (Gonzalez-Ortiz et al., 2023). Similarly, *GZMB* has been identified as a potential drug target to slow brain aging (Yi et al., 2025), showing up in genetic association studies. It has also appeared in neurons and T cells from individuals with neurodegenerative disease and may be involved in the apoptosis of diseased or injured cells, according to murine and *in vitro* studies (Haile et al., 2011; Wang et al., 2012). This emphasizes the clinical potential of isoform studies to identify specific biomarkers.

### Long-read single-cell approaches reveal functional transcriptomic and proteomic differences overlooked in conventional gene-level analyses

While gene-level RNA-seq provides important insights into trends regarding gene expression in disease, it often does not reflect the proteins generated. This is due to the many factors that drive protein abundance and function (e.g., alternative splicing, transcriptional bursting, translation efficiency, protein folding, protein stability) (Liu et al., 2016) which are not currently well understood. A major criticism of transcriptomic studies has been their poor correlation to protein-level abundance and function, which limits biological and clinical translatability. Isoform-level transcriptomics may help to bridge the gap between transcriptomics and proteomics, allowing disease studies to be more efficient and increasingly translatable.

mRNA diversity within individual genes can be vast, with isoforms exhibiting a range of protein-coding potential. Individual isoforms may exhibit truncated UTRs, premature stop codons, missing exons with crucial functional domains, or absent signal peptides required for proper cellular localization. By resolving individual isoforms using long-read sequencing, we can better determine which isoforms are expressed across tissues and cell types, potentially providing more accurate predicted protein expression. Additionally, combining isoform analyses with open-source computational tools (e.g., InterPro (Jones et al., 2014; Blum et al., 2025), AlphaFold2 (Jumper et al., 2021), SAPFIR (Zhou et al., 2022)), enables prediction of differences between structure, function, localization, and translational efficiency, as well as their combined impact on cell types of interest. Our ability to predict changes in functionality on the protein level based on the isoforms expressed, as shown in *GZMB*, may allow us to better predict how elevated gene expression in disease reflects changes in function, and which isoforms would be more likely to be degraded. By performing these analyses, our understanding is enhanced not only regarding the function of protein isoforms within a single gene but also how these isoforms are regulated.

As discussed in our *CD8B* isoform analyses, proteins that have seemingly the same protein sequence may exhibit different expression patterns between cell types. Similarly, our identification of a truncated *GZMB* isoform and secreted *CD3G* isoform suggests that isoforms within the same gene may have different regulatory patterns and functions. These examples highlight the new hypotheses afforded by long-read scRNA-seq; these tools provide mechanistic insights into the relationship between isoform structure and protein function, addressing a critical gap in our understanding of transcriptomic regulation.

Lastly, we identified several isoforms previously unannotated that were not only expressed in a cell-type-specific manner but were the predominant isoform within their gene. These new isoform analyses support the notion that long-read single-cell sequencing combined with isoform discovery informs our knowledge of the isonome on a cell-type-specific basis, which would remain hidden in gene-level analyses.

### Limitations and future improvements

Single-cell datasets are inherently structurally sparse (Lähnemann et al., 2020); some of this sparsity reflects true biological zeros produced by transcriptional bursting, where active production of mRNA from a single gene is turned on and off, producing sporadic expression across cells within the same population (Jiang et al., 2022). Because RNA-seq captures a single snapshot of this process, cells that are sampled when mRNA production is transiently “off” may appear to not to express a gene, despite its capability to do so. Along with biological dropout events, single-cell datasets also exhibit technical dropout, where genes are expressed in a cell but remain undetected, increasing the number of false negatives. This challenge is amplified when combined with long read sequencing, because reads are longer and overall depth is lower, capture inefficiency can produce large numbers of undetected transcripts. The combined effect of transcriptional bursting and technical dropout events inflates the zero counts in these datasets, making it difficult to decipher biological and technical zeros.

Many probabilistic machine learning approaches recommended for scRNA-seq analyses learn latent structure from sparse count data and can incorporate zero-inflated likelihoods (Lähnemann et al., 2020). For example, scVI (Lopez et al., 2018) defaults to a zero-inflated negative binomial (ZINB) (Greene, 1994) distribution likelihood for each gene, adjusting according to cell-specific library depth for estimates of denoised expression. Although scVI has been recognized as a top-performer for atlas-level integration (Luecken et al., 2022), a recent benchmarking study (Valyaeva et al., 2026) reported that scVI is one model that often replaces zeros with very small positive values, which can inflate expression such that, under a >0 definition, every gene is expressed in every cell. In contrast, AutoZI infers zero-inflation for each feature, selectively applying a ZINB component where necessary, rather than applying it uniformly. While utilizing the AutoZI denoised layer can similarly reduce exact zeros, the feature-level inference reduces the risk of improbable expression being inflated broadly. Despite this, AutoZI is not a true replacement for depth. Limited depth can still lead to an underestimation of isoform prevalence within a given cell type, resulting in an exaggerated apparent cell-type specificity for transcripts lowly expressed in a given cell-type due to undersampling. Cell-type-specific patterns with lowly expressed isoforms should therefore be interpreted with caution.

Despite the large number of reads generated per sample, the primary limitation of this study is read depth, given the large number of cells captured (three R10 flow cells across 30,000 cells, per sample). While our achieved read depth was sufficient to analyze the more highly expressed marker genes and associated isoforms, it was not sufficient for broader analyses across genes and isoforms with lower expression. Because reads were distributed across a large number of cells, the resulting count matrix was highly sparse; nearly all genes and approximately half of isoforms (∼99% of genes, ∼48.7% of isoforms, Supplementary Table S2) demonstrated evidence consistent with zero inflation under AutoZI model testing. To account for this sparsity, we modeled counts using a ZINB framework, where recommended by AutoZI. This is important in our dataset on the isoform-level, where not all isoforms require its use.

The major conundrum labs encounter regarding depth in long-read scRNA-seq studies is whether to target fewer cells deeply or sequence more cells at a shallower depth. An alternative to this is to use a targeted approach, focusing on only specific genes of interest; however, this can also bias the analyses toward known isoforms and genes. The Long-Read RNA-Seq Genome Annotation Assessment Project (LRGASP) Consortium released a report from 2024 that stated that longer sequences with lower error rates produced greater accuracy for transcript identification than those with greater read depth; however, deeper and higher-throughput studies produced more accurate quantification (Pardo-Palacios et al., 2024).

To fully leverage the benefits of single-cell long-read sequencing, future studies using this methodology should aim for greater sequencing depth, either by using fewer cells or more flow cells. According to similar studies that obtained greater depth, 10,000-20,000 reads per cell may be considered sufficient for long-read scRNA-seq analyses with normal corrective measures (Shiau et al., 2023). However, the rate of zero-inflated genes and isoforms in these datasets that may be better modeled by ZINB would be worthy of investigation. In future experiments using the same number of flow cells, we recommend reducing the number of cells targeted to 5,000, which we predict will result in a targeted depth of ∼15,000 reads per cell when utilizing 3 flow cells per sample. These recommendations will change if throughput per flow cell increases over time.

Although PIPseq captures transcripts via reverse transcription from the poly(A) tail, we cannot exclude the possibility of internal priming or 3′ RNA degradation producing seemingly truncated transcript models (Webber et al., 2025). Bambu’s conservative framework reduces this risk by prioritizing splice junction structures for the detection of novel isoforms, however we did not specifically evaluate poly(A)-like sequence motifs near the ends of the 126 novel transcripts. Therefore, some of these transcripts may reflect technical artifacts rather than true novel transcript structures.

Lastly, a major limitation of this study lies in the biological context of the optimization dataset. Since this study is composed of a single donor, biological variation between individuals cannot be evaluated. The conclusions produced from this proof-of-concept study are intended to be illustrative, rather than biologically generalizable. Our goal is not to establish population-level conclusions, but to demonstrate a reproducible and biologically grounded analytical framework for single-cell long-read data, while evaluating a novel adaptation to a short-read approach. The findings presented here are intended to validate the feasibility of long-read PIPseq and root single-cell long-read analyses in biology, rather than to provide an exhaustive characterization of PBMCs.

Our knowledge of the isonome and its impact on the proteome remains limited, as evidenced by the genes that produce multiple protein-coding isoforms whose translated sequences and functions are uncharacterized. Though a single dataset can only represent a partial characterization of the isonome, advances in long-read scRNA-seq will provide a more comprehensive view of isoform diversity across cell types. While previous long-read scRNA-seq studies have primarily focused on cataloging isoform diversity, our work extends these efforts by assessing structural differences between isoforms of cell-type-specific markers and linking these to cell-type enrichment and potential functional differences across immune populations. To fully understand the influence of distinct isoforms on biology, researchers must continue to explore innovative approaches to capturing these complex signatures and testing them experimentally. In the future, this framework may also be extended to incorporate aspects of the epitranscriptome with single-cell resolution by integrating with approaches that profile RNA modifications, such as DART-seq detection of m6A, a modification that can impact mRNA stability (Rücklé et al., 2023). These approaches could enhance the field’s understanding of how preferential splicing for different transcripts is regulated by RNA modifications, and which transcripts are likely to carry to the protein level.

The adapted microfluidic-free approach and accompanying computational pipeline described here provide a benchtop-accessible preparation for long-read single-cell sequencing, generating a broad view of the isonomic landscape of PBMCs with one of the greatest numbers of reads and cells produced in a single-cell long-read study to date.

## Methods

For a full list of reagents and materials used, see Supplementary Material.

### Workspace preparation and cleanup

Unless otherwise stated, before and after each day of the protocol, UV light was used in the cell culture hood for 30 min and workspace and micropipettes were cleaned with a 70% ethanol solution and RNase decontaminant solution (*Invitrogen,* #AM9782). Since all steps were performed within an open system, it was imperative to perform all possible steps in this sterile environment to minimize RNase contamination.

#### Day 1: PBMC isolation

PBMCs for this proof-of-principle study were isolated from a blood sample voluntarily donated with informed consent from a 43-year-old healthy Caucasian male. A total of 20 mL of venous blood was collected into two 10 mL K2EDTA anticoagulant tubes (*VWR* #BDAM367525) and transported to the laboratory at room-temperature. Tubes were placed on a rocker for approximately 10 min to resuspend blood cells. Ficoll-Paque PLUS (*Millipore Sigma* #17-1440-020) was prepared according to directions from the manufacturer. PBMCs were isolated using an optimized version of the Sepmate protocol (V2.0.0; *Stemcell Technologies*, #85450). Briefly, room-temperature Ficoll-Paque PLUS was mixed by inversion, and 15 mL was aliquoted into the lower section of a 50 mL Sepmate Tube (*Stemcell Technologies*, #85450). Whole Blood was diluted 1:1 with room-temperature PBMC Medium (1xPBS +2% FBS) and layered onto the Ficoll-Paque PLUS in the Sepmate tube before centrifugation (10 min, 1200xg, 20 °C, max acceleration and break). The top layer (containing PBMCs and plasma) was decanted into a new tube and brought to a volume of 50 mL using PBMC Medium. Samples were centrifuged (10min, 300xg, 4 °C, acceleration 9, break 9) and the supernatant was aspirated to 5 mL to remove the plasma layer. The bottom of the tube was gently flicked to break up the cell pellet, and PBMC Medium was added to 50 mL and centrifuged (8 min, 200xg, 4 °C, acceleration 9, break 1) before removing as much supernatant as possible without disturbing the cells. Red blood cell lysis was performed for 5 min according to manufacturer’s instructions (*Invitrogen* #00-4300-54), then washed with 15 mL 1xPBS (10 min, 900xg, 4 °C, max acceleration and break).

### PIPseq scRNA-seq preparation overview

We modified the PIPseq T20 V4.0PLUS Single Cell RNA Kit User Guide (Revision 8.9) protocol for adaptation to long-read sequencing. Vortexing to mix cell suspensions and sample mixtures is abundant in this protocol; however, since we have found that vortexing is a major source of shearing, we minimized vortexing after cDNA isolation from PIPs to maximize fragment length. Unless otherwise stated, any time a wash occurred or a cell pellet was formed from centrifugation, gentle tapping of the tube was used to dissociate the pellet before resuspending and pipetting to mix gently and slowly with a wide-bore pipette tip. The PIPseq protocol was performed over 3 days with our noted alterations, as indicated below. The base protocol includes 5 stop points ranging from overnight to 96 h where users can stop if needed; however, we chose to utilize only 3 stop points and perform the protocol on consecutive days to minimize RNA/cDNA degradation from extended periods of storage. We also did not store cDNA at temperatures below 4 °C until after sequencing to avoid shearing during multiple freeze-thaw cycles.

As recommended in the protocol, when it was noted that we “spin down” samples, this was done on a benchtop minifuge reaching speeds up to 20,000RPM. All plastic tubes and reagents described in the PIPseq sections below were included in the kit, unless noted. A swinging bucket rotor was used for PIPseq to minimize damage to cells, RNA, and cDNA, as suggested (*Thermo Scientific,* #755007210). Unless otherwise mentioned, all reagents and plastics in the PIPseq section of the protocol originated from one of the following kits that make up the T20: (PIPseq T20 3′ Single Cell RNA Ambient Kit v4.0PLUS (*Fluent* #FBS-SCR-T20-4-V4.05-1), PIPseq T20 3′ Single Cell RNA 4 °C Kit v3.0 or 4.0 (*Fluent* #FBS-SCR-T20-4-V3&V4-2), PIPseq T20 3′ Single Cell RNA 20 °C Kit v4.0PLUS (*Fluent* #FBS-SCR-T20-4-V4.05-3), PIPseq T20 3′ Single Cell RNA -80 °C Kit v4.0PLUS (*Fluent* #FBS-SCR-T20-4-V4.05-4), PIPseq T20 3′ Single Cell Consumables Kit v4.0PLUS (*Fluent* #FBS-SCR-T20-4-V4.05-6)).

#### Day 1: PIPseq—cell preparation

Immediately after isolating PBMCs and washing cells in 1xPBS, rather than performing a single wash in warmed Cell Suspension Buffer, samples were washed twice in 1 mL ice-cold PIPseq Cell Suspension Buffer (one at room temperature, one at 4 °C) to ensure the gentle removal of reagents inhibitory to PIPseq (e.g., FBS). Cells were centrifuged at a setting we optimized for most cell-types (6min, 300xg, 4 °C) and supernatant was aspirated carefully. We recommend that users tailor the temperature and time of centrifugation to their cell-type or tissue of interest based on the optimal conditions for survival. A swinging bucket with a 1 mL adapter is gentler on the cells during this section, aiding in better viability. Between washes, the cell pellet was dissociated by gently tapping the bottom of the tube and cell suspension was mixed with slow pipetting with wide-bore tips to reduce mechanical strain. We found that mixing with a wide-bore tip alone, as recommended in the base protocol, was insufficient to obtaining a uniform cell suspension. Dissociating the pellet with gentle tapping replaced the recommended need to increase pipetting force, which may increase mechanical strain on cells and result in lysis. After washes, the cell pellet was resuspended in 500 µL of PIPseq Cell Suspension Buffer (instead of 200–400μL, again to reduce the effect of any residual FBS), as described above (gently tapping and wide-bore mixing). Cells were counted using trypan blue (*PromoKine* #PK-CA902-1209) to determine viability and cell concentration (Formula 1). Since PIPseq has an estimated 50% capture rate, to target 30,000 cells for sequencing, we identified that a total of 60,000 cells would be needed in a volume of 8 μL for the PIPseq protocol. Thus, our Targeted Concentration was 7,500 cells/µL for loading. The Targeted Concentration was then used to determine the volume of Cell Suspension Buffer to add to the sample for the desired concentration (Formula 2). If the value produced from Formula 2 was negative, we removed that volume of supernatant rather than adding to reach the Targeted Concentration.

$$Cells/µL=\left(\frac{total\;live\;cell\;count}{Number\;of\;fields\;counted}\right)*\;10*\;1mL*\;Dilution\;Factor$$ (1)

$$Volume\;Buffer\;to\;add=\left(Volume\;at\;counting\right)-\frac{\left(Cells/µL\right)*\left(Volume\;at\;counting\right)}{Targeted\;Concentration}$$ (2)

#### Day 1: PIPseq—cell capture and lysis

To adjust cell suspension concentration, samples were centrifuged at our optimized setting (6 min, 300xg 4 °C, max acceleration and break) and the volume was adjusted according to the value calculated with Formula 2. Cell counts (Formula 1) were repeated to ensure accurate concentration and >90% viability before loading into tube containing PIPs. As in the base protocol, the cell suspension was gently pipette-mixed with a wide-bore tip before transferring 8 µL of cell suspension and 2 µL of SUPERase•In RNase Inhibitor (20U/µL) (*Invitrogen* # AM2696) into the PIP mixture. The cell:PIP mixture was very slowly pipette-mixed using a standard-bore low-retention P200 pipette as recommended by the protocol. We ensured that very slow, even strokes were used, that the emulsion was released along the inner side wall of the tube, and that the pipette was not pushed to the second stop until mixing is complete. Although this protocol recommends the use of a multi-channel pipette while mixing the cell:PIP mixture, we found using a single-channel pipette reduced the amount of bubbles and foam produced during this step, a crucial problem given how viscous and prone to foaming the solution is. Although a small number of bubbles is fine, if excessive foaming occurred, we briefly centrifuged on the minifuge to remove. Excessive centrifugation can cause the removal of cells from PIPsand was used sparingly. On the Fluent Website, a video protocol of this step was previously included at the beginning of the Cell Capture step in the “Legacy: PIPseq T20 V4.0PLUS Single Cell RNA Kit User Guide” (Revision 8.9). This video tutorial can now be found on the Illumina website under “Illumina Single Cell 3′ RNA Prep, T20 Workflow” (https://support.illumina.com/sequencing/sequencing_kits/illumina-single-cell-prep/training.html). We highly recommend users watch this video and take note of the images throughout the protocol before applying the changes made for long reads.

The remainder of this section follows the standard v4.0PLUS PIPseq protocol as written. Briefly, Partitioning Reagent was added before vortexing on the PIPseq rotating vortexer. After vortexing, the bottom phase of the sample, containing Partitioning Reagent Waste, was removed to the lowest line of the blue PIPseq tube stand. Partitioning reagent and Chemical Lysis Buffer 3 (CLB3) were combined and vortexed before adding to the PIP emulsion. The PIP emulsion was then gently inverted to mix ten times. Cells were lysed using setting A (Table 2) in the PIPseq Dry Bath (*Fluent* #FBS-SCR-PDB) and held at 15 °C–25 °C overnight:

**TABLE 2:** PIPseq dry bath setting a parameters for heat-induced cell lysis.

Temperature	25 °C	After preheating, press “Skip”, then “Yes”	25 °C	37 °C	25 °C	15 °C–25 °C
Time	Hold		15 min	45 min	10 min	HOLD

These settings were performed using a dry bath, but a thermocycler can also be used to induce cell lysis.

#### Day 2: PIPseq—mRNA isolation

This section was performed according to the base protocol. Briefly, after incubation, the bottom phase was removed to the middle line on the blue PIPseq tube stand. Breaking Buffer and De-partitioning reagent were both added to the sample and the sample was inverted gently 10 to 20 times, then spun down briefly. Although the PIP formulation is designed to protect samples from damage during vortexing, our experience adapting this protocol suggests that this protection is inconsistent for long strands. The bottom, pink-colored waste layer was removed carefully to avoid disturbing PIP layer and PIPs were transferred to an aliquot of Washing Buffer. A total of three washes were performed as follows: 1) The pellet was washed with 12 mL Washing Buffer, 2) gently dispersing the cell pellet by tapping and inverting the sample tube, 3) centrifuging in a swinging bucket rotor (2 min, 750xg, 4 °C, low break and max acceleration), and 4) slowly aspirating the supernatant without disturbing the pellet. The PIP mixture was transferred to a pre-weighed 1.5 mL tube and weighed to calculate the volume that needed to be removed to normalize the mixture to 250 µL (Formula 3). Samples were spun down ∼30 s before carefully removing the determined volume from Formula 3.

$$\text{Volume to remove}=\left(\text{Total mass }–\text{ Tube mass}\right)\;–\;250\;uL$$ (3)

#### Day 2: PIPseq—cDNA synthesis

A master mix was prepared with the following recipe per sample: 232.2 µL RT Additive Mix, 21.6 µL TSO, 16.2 µL RT Enzyme Mix. The pellet was dissociated by gently tapping the bottom of the tube, 250 µL of the master mix was added, and the tube was gently inverted twenty times to mix. Protocol “C” on the PIPseq Dry Bath was used for cDNA synthesis (lid = 105°) (Table 3).

**TABLE 3:** PIPseq dry bath setting “C” parameters for cDNA Synthesis.

Temperature	25 °C	42 °C	85 °C	4 °C
Time	30 min	90 min	10 min	Hold

#### Day 2: PIPseq—cDNA amplification

Rather than storing the samples at 4 °C overnight, after cDNA synthesis, we proceeded directly to the cDNA Amplification steps. Samples were spun down for 30 s and 300 µL of supernatant was removed. PIPs were washed three times in 0.5X Wash Buffer according to the standard protocol, including brief vortexing (5s, 3000RPM, PIPseq vortexer). Aspiration volume to normalize each mixture volume to 250 µL was again calculated (Formula 3). Samples were spun down for 30 s, and the designated volume of supernatant was removed. WTA Mastermix was generated according to the following recipe per sample: 94.4 µL 4X PCR Master Mix and 1.87 µL WTA Primer. 85.7 µL of WTA Mastermix was added to each sample and sample mixtures were vortexed briefly (5s, 3000RPM, PIPseq vortexer). The WTA reaction mixture was distributed into eight 42 µL aliquots across a PCR tube strip. Samples underwent amplification (lid = 105 °C), then the samples were stored at 4 °C in the thermocycler overnight (Table 4). 15 cycles for the step that includes both annealing and extending were chosen according to the recommended conditions for 30,000 primary cells.

**TABLE 4:** Thermocycler settings for cDNA Amplification.

Temperature	Time	# of cycles
95 °C	3 min	1
98 °C 69 °C	15s 4 min 20 s	15
72 °C	5 min	1
4 °C	Hold	—

#### Day 3: PIPseq—isolate cDNA from PIPs

To split open PIPs, 75 µL of CE Buffer was added to each WTA reaction, quickly tapped 5 times on the vortexer (3000RPM) and spun down 30 s, instead of 5 s, to ensure tight pelleting of PIPs off tube walls. WTA products were pooled by sample and tap-vortexed again five times. Supernatant containing cDNA was collected and prepared for SPRI bead enrichment at a magnetic bead ratio of ×0.8. Rather than vortexing samples and incubating for 5 min as stated in the base protocol, samples were gently flicked to mix and incubated on the hula mixer for 10 min at room temperature. This change was made to minimize shearing on the isolated cDNA, while ensuring samples are thoroughly mixed and beads did not settle. After incubation, the sample tubes were bound to a magnet (*Invitrogen* #12321D) for 5 min and washed twice with 85% ethanol for 30 s each. Beads containing cDNA were resuspended in room-temperature IDTE (pH 8.0), gently mixed by pipetting with a wide-bore tip, and incubated on a hula mixer for 5 min. Sample tubes were bound to the magnet for 2 min and, using a wide-bore pipette tip, cDNA was collected into a 1.5 mL LoBind Eppendorf tube.

The PIPseq protocol utilizes the included TE Buffer (pH 8.0) to collect cDNA from beads, however we decided to utilize IDTE (pH 8.0) instead. IDTE has a lower concentration of EDTA, which can act as a chelating agent capable of inhibiting PCR, so ONT recommends ensuring that input cDNA have low levels of similar contaminants. For this reason, we chose IDTE over TE buffer.

### Quality control (QC)

QC Checks were performed after the completion of the PIPseq (Day 3) and ONT (Day 4) portions of the protocol. The 1X dsDNA Qubit High Sensitivity Kit (*ThermoFisher* #Q33231) was used to determine cDNA concentration, with the following deviations: (1) 1 μL of the sample was diluted in 9 μL Qubit Working Solution before being sonicated for 3 min to break up long strands of cDNA for accurate concentration measurement; (2) 190 µL of Qubit Working Solution was added to each Qubit sample tube (*Invitrogen #Q33252*) for a final volume of 200µL, then vortexed for three to 5 seconds; (3) the sample was spun in the benchtop minifuge briefly before incubating at room temperature for 10 min. After incubation, the standard protocol for the kit was used to determine concentration using the Qubit (*ThermoFisher Scientific* #Q33327). Based on the concentration, the cDNA was diluted to fall below 500 pg/μL in alignment with the maximum detectable concentration for the standard Femto Pulse gDNA 165 kb protocol (*Agilent*, #FP-1002-0275). The Femto Pulse gDNA 165 kb protocol was used to determine that fragments were largely intact and demonstrated an average amplicon size >500bp (Supplementary Table S7). The Standard PIPseq protocol utilizes a TapeStation 4200 for the QC assessment; however, we have not tested the reliability of this method for our described preparation.

### Custom oligos for adaptation of PIPseq cDNA for ONT sequencing

Custom DNA oligos were designed to provide overlapping sequences that allow for the connection of cDNA primed with the PIPseq WTA Primer to the ONT-specific cDNA primer (cPRM). These oligos bridged the PIPseq protocol to the ONT Single-Cell Protocol and allowed the nanopore sequencing of cDNA produced via PIPseq. The Forward primer has a Biotin tag on the 5′ end, indicated by “5Biosg”, which allows for magnetic enrichment of strands that are compatible with ONT sequencing. Oligos with the below sequences were ordered from Integrated DNA Technologies (IDT) at 100 nM (dry) with HPLC Purification. Once received, oligos were reconstituted in TE Buffer (pH 8.0) at a stock concentration of 100 µM and stored at −20 °C. Stock concentrations were diluted in TE Buffer to a working concentration of 10 µM for the protocol. Oligo sequences in Table 5 are split according to the portion of the sequence it aligns to.

**TABLE 5:** Custom oligo sequences.

Oligo name	Sequence portion	Sequence
[Btn]Fwd_3580_ partial_read1_PIP	Biotin tag	5’ -/5Biosg/
	Overhang	CAGC
	ONT-specific cDNA primer (cPRM)	ACT​TGC​CTG​TCG​CTC​TAT​CTT​C
	PIPseq WTA primer	CTCTTTCCCTACACGACGCTC −3′
Rev_PR2_partial_ TSO_PIP	Overhang	5’ - CAGCT
	ONT-specific cDNA primer (cPRM)	TTC​TGT​TGG​TGC​TGA​TAT​TGC
	PIPseq WTA primer	AAG​CAG​TGG​TAT​CAA​CGC​AGA​G - 3′

#### Days 4 and 5: ONT library preparation and sequencing

This section was adapted from two protocols provided by Oxford Nanopore Technologies (ONT): “*Single-cell transcriptomics with cDNA prepared using 10X Genomics*” and “*cDNA-PCR Sequencing V14 (SQK-PCS114)*”. Because of a legal agreement with ONT, we are unable to disclose the specific steps of these protocols. For clarity, these will be referred to as “s*ingle-cell ONT protocol*” and “*V14 protocol*”, respectively, throughout this section.

Briefly, the single-cell ONT protocol used PCR to attach custom oligos, which ensured compatibility of the PIPseq-generated cDNA with the ONT cPRM. Biotin-mediated size selection was used to enrich for library molecules containing the custom oligos. PCR was then performed to attach the isolated transcripts to the ONT cDNA Primer (cPRM) before performing a quality check, as described in the Quality Control (QC) section. The next day (Day 5), the adapter containing the motor protein was attached to each strand and the sample was slowly pipetted into flow cells in the PromethION. See Figure 1c (middle box) for the final structure of reads before sequencing.

We made the following changes to these published protocols: Any time either ONT protocol called for the cDNA sample to be vortexed, we instead pipetted very gently 10-20 times with a wide-bore pipette tip to mix. The ONT single-cell method was designed to be used with single-cell barcoded cDNA produced with the 10X Genomics Next GEM Single Cell 3′ Kit (V3.1). Our modifications to this protocol utilize cDNA amplicons produced using the long-read PIPseq (V4.0PLUS) protocol above. As such, we recommend the modified sequences for the custom oligos described in the Custom Oligos for Adaptation of PIPseq cDNA for ONT Sequencing methods section, which were designed to bridge the ONT and PIPseq sections of the protocol to ensure compatibility. The modifications detailed in this protocol weredesigned with the updated ONT R10 chemistry in mind. Sequencing preparation for the single-cell ONT protocol was intended to be performed using the PCR-cDNA Sequencing Kit (*ONT, #SQK-PCS111*) with PromethION R9.4.1 flow cells (*ONT #FLO-PRO002*). Our protocol utilizes the updated ONT PCR-cDNA Sequencing Kit V14 (*ONT #SQK-PCS114*) with PromethION R10 flow cells (*ONT, #FLO-PRO0000*). The standard protocol uses an AMPure XP bead ratio of 0.8X for both the “Pre-Pull-Down” and “Post-Pull-Down” bead enrichment steps; however, our lab found that using a ratio of 0.7X enriches for larger fragments. Bead washing for the “Pull-down” section of the protocol (steps 1-7 in single-cell ONT protocol, “Pull-down” section), was performed during the pre-pull-down PCR cycling to increase efficiency. Aside from these alterations, the biotin-tagging reaction and pre-pull-down PCR sections were performed as written in the published single-cell ONT protocol. Targeting three flow cells, we aimed to recover >210 ng cDNA before sequencing. After Post-Pull-Down, QC was performed and concentration was assessed using the protocols for the Qubit High Sensitivity Kit and Femto Pulse gDNA 165 kb kit, as described in the Quality Control (QC) methods section.

The next day, once fragment quality had been confirmed, the sequencing preparation was resumed. Starting at the “Adapter Addition” section of the ONT single-cell protocol, we followed the adapter addition, priming, and flow cell loading steps contained within the V14 protocol. The average amplicon size from the Agilent Femto Pulse Bioanalyzer was used to calculate the required sample volume for 70 fmol and diluted into 31 µL of Elution Buffer (EB). Just before loading the samples into the flow cells, the sample tubes were gently flicked to mix. We waited a minimum of 20 min after loading the library into the flow cells before initiating sequencing. Sample cDNA libraries were sequenced continuously until the end of the flow cell life. Data were collected using MinKNOW (23.11.4) and. fast5 files were basecalled using the Dorado (7.2.13+fba8e8925) graphics processing unit (GPU) basecaller. Any remaining sample was placed at −80 °C for long-term storage.

### Computing power used for analyses

Analyses were performed using the University of Kentucky Morgan Computing Cluster (MCC), a batch-processing cluster with AMD EPYC Rome processor cores distributed across 182 compute nodes. Each node is configured with 512GB to 4 TB of memory. The MCC has a shared GPFS parallel filesystem with 3 PB of disk storage for user home, project, and scratch directories. Additionally, the cluster has a high-speed 10 Gb data transfer node for efficient data movement to and from external servers. Analyses were performed in Jupyter Notebook (Kluyver et al., 2026; Granger and Perez, 2021) using Python3 v3.10.13.

### Read preprocessing and barcode rescue

After sequencing, the resulting files were moved off the PromethION Data Acquisition Units (*ONT, #PRO-PRCA100*) to our storage units. The sequencing summary .txt files and the passing FASTQ files were copied onto one of the University of Kentucky’s computing clusters. All FASTQ files generated from Dorado (V7.2.13+fba8e8925) were processed in batches of 500 files separated by sample and flowcell, then concatenated into a single file by flow cell. Using a Python 3.10.13 script, reads with a mean base quality less than nine were removed (See Data and code availability section). The resulting filtered FASTQ was run through pychopper (2.7.10) using custom primers: The Fwd_3580_partial_read1_PIP sequence minus the overhang sequence and the cPRM from the Rev_PR2_partial_TSO_PIP sequence. The rescued fused reads from pychopper were concatenated with the regular reads output from pychopper. The number of reads was counted and saved, and the files were split into multiple files of 8,000 reads each.

Due to the higher error rate in long-read data, simply running paired-end reads through PIPseeker was insufficient for barcode rescue. PIPseeker (Clark et al., 2023) uses Hamming distance (Hamming, 1950) for barcode matching and error correction, which accounts only for base substitutions, the most common error in short-read sequencing. While insertion and deletion errors are rare in short-read sequencing (Schirmer et al., 2015), long-read sequencing frequently produces insertions and deletions, with an expected insertion rate ∼0.46%, deletion rate of ∼0.62%, and mismatch rate ∼2.1% for ONT-based sequencing (Zhang et al., 2023). This may shift the sequence of the barcode, so with the tiered structure of the barcode, a more complex pre-processing approach was needed before plugging the data into PIPseeker. Unlike Hamming distance, Levenshtein distance (Levenshtein, 1966) accounts for insertions, deletions, and substitutions, rather than just substitutions alone. Adopting an approach utilizing Levenshtein distance enhances the accuracy and reliability of barcode recovery.

Java code (openjdk 17.0.13) was used on the pychopper output to rescue barcodes and convert them into pseudo-paired-end read files for PIPseeker (Clark et al., 2023), as described below. During the rescue process, each read was examined to find the tiered barcode used by PIPseeker. We then calculated the Levenshtein distance between each tier entry and every whitelist sequence for that tier. If an exact match for the tier was not found, (i.e., a Levenshtein distance of 0) we performed one of three actions: (1) If the lowest Levenshtein distance was greater than two, the read was discarded, as we could not determine the correct sequence for the tier; (2) if two or more correct sequences shared the lowest Levenshtein distance in the tier, the barcode was labeled as ambiguous and sent to a separate file to attempt rescuing later; (3) the tier sequence was corrected to the whitelist sequence with the lowest Levenshtein distance.

PIPseeker (Clark et al., 2023) is designed for use with short-read data that sequences libraries using paired-end reads. As required, the read was converted into pseudo-paired-end read files, with a Read 1 (R1) file containing the corrected barcode sequence indicating that of an individual cell and a molecular index sequence (UMI) unique to each mRNA molecule. A Read 2 (R2) file contained the cDNA contents from the mRNA captured in each PIP.

A list of all full-length barcodes in the R1 file was kept to use as a sample-specific master whitelist of barcodes seen from that sample. All barcode whitelists across batches were combined into a single master whitelist, split by sample. This whitelist was then fed into a second script (java) to rescue barcodes, which compared the ambiguous barcodes in the reads to these sample whitelists. Unambiguous barcode tiers were used to select the potential correct barcodes from the sample whitelist. The ambiguous tiers were then compared to the tier values of those potential barcodes. If using this whitelist made the barcode unambiguous (i.e., there was now only one tier barcode that had the lowest Levenshtein distance to the ambiguous tier), the read was kept and converted to a pseudo-paired-end read as described above, otherwise, the read was discarded.

We developed this approach to rescue as many reads as possible while being extremely conservative to avoid introducing false signals into the data. As an assessment of the worst-case scenario, exactly 11,414,734 and 9,203,793 reads were determined to have ambiguous barcodes in at least one tier for PBMC1 and PBMC2, respectively; all of these reads were set aside for the second round of rescuing. Of the ambiguous reads, only 1,112,557 (9.7%) and 857,204 (9.3%) met our stringent criteria to be rescued because they were within a Levenshtein distance of 2*[n ambiguous tiers]-1 with another barcode that we had already seen confidently. Of the total number of reads ultimately included before PIPseeker for PBMC1 (158,309,133) and PBMC2 (103,099,471; see Supplementary Table S1), the rescued reads only constitute 0.70% (1,112,557/158,309,133) and 0.83% (857,204/103,099,471) for PBMC1 and PBMC2, respectively. Thus, as a worst-case scenario, if we assume that all reads that were ambiguous in the first round were ultimately assigned to the wrong cell barcode (highly improbable), their effects would still be minimal.

We then ran the pseudo-paired-end FASTQs through PIPseeker (v02.01.04) (Clark et al., 2023), which replaced barcodes with a shorter version and applied additional read filtering. Software documentation can be read for full details; however, briefly, the TSO and poly-A sequences were removed from the R2 files, and a FASTQ file was exported from reads greater than 20 nt with barcodes and UMI sequences in the read header. Reads were then concatenated by sample and flowcell into one FASTQ file (no longer paired-end) to use later for isoform discovery. Reads were also demultiplexed into separate FASTQs (no longer paired-end) by barcode and isoforms with at least 100 reads per cell were kept for single-cell isoform quantification.

### Pseudo-bulk alignment and isoform discovery

Our process for genomic alignment and quality control was fundamentally the same as that described in Heberle et al. (2023), in the section titled “Read preprocessing, genomic alignment and quality control”. Briefly, reads were preprocessed to find full-length molecules, and adapter and primer sections were trimmed, then aligned to the GRCh38 human genome using minimap2 with splice-aware settings enabled. Reads with a MAPQ score <10 as well as secondary and supplementary alignments were excluded. Deviations from the described process included earlier use of pychopper, as described above, and the use of updated versions of minimap2 (Li, 2018) (v2.28-r1209) and SAMtools (Li et al., 2009) (v1.19.2). Please see “Data and Code Availability” section for more information.

Our process for isoform discovery and quantification was also similar to that reported in the first paragraph of the section titled “Transcript discovery and quantification” in Heberle, *et al* (Heberle et al., 2025). Briefly, filtered BAM files were utilized for transcript identification and discovery and quantification with Bambu, using the reference genome and annotation file. The changes are listed here: ERCC spike-ins were not used, since these samples were within the same batch. We used the Ensembl HG38 release 113 annotation file. An updated version of Bambu (Chen et al., 2023) (v3.8.1) was used instead of v3.0.5. We used the New Discovery Rate (NDR) recommended by the Bambu machine learning model, which was 0.051 for this dataset. Using the outputted extended annotation file, we ran gffcompare (Pertea and Pertea, 2020) (v0.12.6) against the Glinos et al. (Glinos et al., 2022), Leung et al. (Leung et al., 2021), and Heberle et al. (Heberle et al., 2025) annotations.

### Single-cell alignment and isoform quantification

Barcode-separated FASTQ files were aligned using minimap2 (Li, 2018), and the aligned BAMs were filtered using SAMtools (Li et al., 2009), as in the pseudo-bulk alignment. However, discovery to find new isoforms was not repeated on the single-cell-level; instead we used the extended annotation from the pseudo-bulk isoform discovery as the annotation passed to Bambu to quantify isoforms. We also batched the FASTQs into groups of 300 files and ran those through the quantification process. After, we merged the matrices outputted by Bambu into a single matrix per output type (gene counts, transcript counts, transcript CPM, full length counts, and unique counts).

### Data processing and bioinformatic quality control

To facilitate file management, initial filtering utilized a java script that automatically removed cells with fewer than 100 reads, and genes expressed in fewer than 10 cells. This pre-filtering was performed due to the immense computational power required to process the raw files, which did not allow loading of the raw files into a Jupyter notebook. Our lower threshold for reads per cell was chosen to be 100 reads, so enough empty droplets were removed to reduce the file size while retaining a sufficient amount to perform SoupX for ambient RNA detection. A lower threshold of 10 cells per gene was chosen in alignment with a benchmarking study used to evaluate the performance of long-read single-cell approaches (Deng E. et al., 2025). After initial filtering to remove empty droplets, supervised filtering was performed to identify appropriate thresholds for low-quality or stressed cells. Cell counts were taken initially and after filtering. QC metrics were computed as described below, and the distribution of each measure was plotted. Ranges for quality threshold were determined based on recommended values (Osorio and Cai, 2021), and then refined using dataset-specific distribution analysis. QC metric distributions were examined using violin and density plots to find transition points separating low-quality droplets from the healthy cell population. Thresholds were set at these inflection points rather than using a fixed quartile. Different sample datasets within the same study underwent QC filtering individually before concatenation. Hemoglobin and mitochondrial genes were identified according to gene prefix, and the following QC metrics were enforced: cells per gene/isoform, total number of genes/isoforms per cell, total transcript counts in a cell, log-transformed total counts for genes/isoforms, percent counts from mitochondrial genes, and percent counts from hemoglobin genes. These metrics (Supplementary Table S2) were employed to identify and dispose of low-quality, stressed, or dying cells. For example, we applied a mitochondrial upper threshold of 15%, adjusted based on the distribution observed in violin plots of mitochondrial gene percentages. We tailored this threshold to be appropriate forboth datasets to ensure that only healthy cells are retained for analysis.

Datasets were converted to AnnData (v0.11.3) (Virshup et al., 2023; Virshup et al., 2024) files to ensure compatibility with downstream packages and combined with an “inner” join to keep only genes common between the datasets. After combining datasets, as a secondary measure to remove doublets, the Scrublet package (v0.2.3) (Wolock et al., 2019) was used to estimate and remove possible doublets. Automatic thresholds for the doublet score for each dataset were calculated at 0.648 (gene) and 0.642 (isoform), however, a more stringent threshold of 0.3 (based on the point of distribution curve plateauing) was used to maximize doublet removal. Summary statistics by sample after filtering can be found in Supplementary Table S8.

### Ambient RNA contamination assessment

To identify whether significant ambient RNA contamination in cell droplets contributed to our isoform expression patterns, we applied the SoupX package (v1.6.2) (Young and Behjati, 2020). An ambient RNA profile was constructed from low-count droplets containing 100-500 total read counts (n = 31,336, median reads = 150) in the post-JavaScript filtering dataset to inform cell-type-specific contamination rates. Cluster annotations were used to derive cluster-informed contamination estimation in the filtered dataset. The estimated global contamination fraction was approximately 1.1%, with no major deviation by cell-type. SoupX-corrected count matrices were used for validation of findings; downstream AutoZI modeling was performed on the filtered uncorrected count matrix.

### Model building and AutoZI

The AutoZI model from the scVI-tools library (V1.2.0) (Clivio et al., 2019; Gayoso et al., 2022) was used to analyze the concatenated data, with batch correction applied using sample metadata. Model hyperparameters (epoch number, learning rate, patience interval, weight decay, latent dimension number and dropout rate) were tuned using training and validation ELBO (evidence lower bound) curves on a 1000-cell subset of the dataset. Candidate parameter sets were then applied to the full dataset and evaluated again for overfitting. ELBO represents the optimization of modeling during variational inference, so these curves were used to assess model convergence and identify evidence of overfitting for each set of parameters. Final parameters were selected according to stable convergence of both training and validation and minimized divergence between them, using the full dataset. The parameter set with the least evidence of overfitting was utilized for our analyses.

The model was initialized with 20 latent dimensions and a dropout rate of 0.3. The model was trained on 90% of the data for a maximum of 300 epochs at a learning rate of 0.01. To minimize over-training, early stopping was enabled (patience = 70) and a weight decay rate of 0.0005 was utilized to prevent the overuse of any one neuron during modeling. The remaining 10% of the dataset was used for training validation. This sampling was performed randomly. After modeling, the latent representations and denoised data were extracted, normalized per 10,000 reads, and stored in the AnnData object to correct for zero inflation. The zero-inflated probabilities were identified and printed for the user’s evaluation, to show the extent of correction needed (Supplementary Table S3).

### Cell clustering and cell-type assignment

With the denoised data from the AutoZI model, clustering was performed using a k-nearest neighbor (KNN) (Cover and Hart, 1967) graph, built with 20 neighbors per cell. Uniform manifold approximation and projection (UMAP) (McInne et al., 2018) was applied for dimensionality reduction, and clusters were identified using the Leiden (Traag et al., 2019) algorithm. The Leiden algorithm (flavor = igraph (Csárdi and Nepusz, 2006; Antonov et al., 2023; Csárdi et al., 2025)) was run at multiple resolutions (0.06 for cell-type, 0.26 for sub-cell-type) to identify clusters and determine the resolution that best represents the biological dataset.

After clustering, cell-type clusters were assigned using known cell-type-specific flow cytometry markers, including surface, secreted, and transcription factor markers. Megakaryocytes, Monocytes, T cells, B cells, and Natural Killer (NK) Cells were detected based on cluster-specific expression of the markers in Supplementary Table S4 and explained further in Supplementary Material. T cell sub-types were detected in clusters that express the broad cell markers (e.g., *CD3D*, *CD3E*, *CD3G* along with the subtype-specific markers. Since RNA expression is often an imperfect proxy for protein abundance due to differences in transcriptional bursting and protein stability (Liu et al., 2016), we tested a range of highly specific markers to assign cell identity. The final markers that we used to determine cell type based on high co-expression in a single cluster are in Supplementary Table S4.

Resolutions with distinct clustering and multiple marker genes that fell within the confines of our biological relevance thresholds were deemed to be most relevant to the study. Markers were also plotted to demonstrate expression levels across the cluster, to ensure that they were widely expressed within the cluster of interest. Marker genes were plotted across clusters to check for high cluster specificity and expression within the cluster of interest, as well as on the pseudobulk level to ensure high raw read count (Supplementary Figure S4).

### InterProScan

Protein sequences from Supplementary Table S6 were annotated with protein domains and features using the InterProScan database (web-based, v5.75-106.0, default settings) (Jones et al., 2014; Blum et al., 2025). Annotations from participating databases (e.g., Pfam (Sonnhammer et al., 1997; Finn et al., 2016; Paysan-Lafosse et al., 2025), SMART (Ponting et al., 1999; Letunic and Bork, 2026), PROSITE (Sigrist et al., 2002; Sigrist et al., 2005; de Castro et al., 2006; Hulo et al., 2008; Sigrist et al., 2026)) were used to distinguish functional differences between protein isoforms according to the domains, families, and functional sites conserved within the protein sequence.

### Sherbrooke alternative protein feature identificatoR (SAPFIR)

To visualize differences in functional features across *CD3G* and *GZMB* isoforms, the Single Gene Annotation function of the Zhou et al, 2022 web server was used, with default settings. The enabled databases included: ProSiteProfiles (Sigrist et al., 2002; Sigrist et al., 2005; de Castro et al., 2006; Hulo et al., 2008; Sigrist et al., 2026), ProSitePatterns (Sigrist et al., 2002; Sigrist et al., 2005; de Castro et al., 2006; Hulo et al., 2008; Sigrist et al., 2026), CDD (Marchler-Bauer et al., 2017; Lu et al., 2020), SMART (Ponting et al., 1999; Letunic and Bork, 2026), Pfam (Sonnhammer et al., 1997; Finn et al., 2016; Paysan-Lafosse et al., 2025), and Gene3D (Yeats et al., 2008; Lees et al., 2010). SAPFIR outputs were used in conjunction with InterProScan to visually summarize isoform-specific differences in features shared between the two sites.

### Conserved domain database (CDD) annotation

To validate InterProScan results showing domains in select proteins, our protein sequences of interest were input directly into the Batch Web CD-Search Tool (Wang et al., 2023; Marchler-Bauer et al., 2017; Lu et al., 2020), with default settings (Expect value threshold = 0.01; maximum number of hits = 500; composition-corrected scoring enabled; low-complexity filter disabled; retired sequenced included). CDD results were used to check for conserved specific and family domain hits, immunoglobulin structural motifs, and heterodimer interfaces. These results contributed to the visual summation of isoform-specific differences.

### Transmembrane helix prediction via DeepTMHMM

To validate the absence of transmembrane domains in our proteins of interest, we used the web server-based DeepTMHMM (v1.0.44) (Hallgren et al., 2022) deep learning model designed to predict and classify transmembrane topology. The results of DeepTMHMM detailed each residue from the protein structure and the location within the cell (e.g., signal peptide, inside cell, alpha membrane, periplasm, outside cell). It also predicted the number of transmembrane domains, to validate InterProScan results.

### Protein structure prediction

Protein structures were predicted for isoforms according to amino acid sequence using the ColabFold v1.5.5 (Mirdita et al., 2022; van Kempen et al., 2023; Eastman et al., 2017) running AlphaFold2-ptm (Jumper et al., 2021). Predicted structures were generated and ranked using the following settings: Multiple-sequence alignments (MSA) were generated using MMseq2 (Mirdita et al., 2019) with the mmseqs2_uniref_env option (Mitchell et al., 2019; Mirdita et al., 2017). The pdb100 database (Berman et al., 2003; Steinegger et al., 2019) was used to source structural templates from known homologs. Modeling was performed with dropout enabled across 8 random seeds, each producing 5 protein model predictions. Protein structures were refined via 12 recycling iterations (feeding the output back into the model) without early stopping (recycle_early_stop_tolerance = 0.0). A greedy pairing strategy, which pairs any taxonomically matching sets, was enabled to align taxonomically matching sequences, and paired MSAs were generated in unpaired_paired mode to allow independent and co-evolutionary alignment. A maximum number of paired:unpaired MSA sequences was set to 256:512. All 5 models per seed underwent AMBER relaxation (Eastman et al., 2017) after generation to improve predicted geometry. Model quality was assessed using the predicted local distance difference test (pLDDT) and predicted aligned error (PAE). The top-ranked model was visualized and colorized according to IDDT. Protein sequences used for modeling can be found in Supplementary Table S6 and outputs including. pdb file for 3-D visualization can be found on Zenodo (Ebbert et al., 2025), as described in Data and code availability.

### Untranslated region (UTR) annotation

Differences in 3′ and 5′ UTR region and length were visualized using the UTRdb 2.0 web server (Lo Giudice et al., 2023), with default settings (UTR_type = 5′3′UTR; organism = Homo_sapiens.GRCh38.107). The Homo_sapiens.GRCh38.107 option was selected under “organism” and the gene symbol of interest was selected. These results were used to determine differences between transcripts with unique expression signatures despite appearing to encode for the same protein.

### Prediction of coding potential of novel transcripts

To evaluate the coding status of novel transcripts from new and known genes, transcript sequences were submitted to the Coding Potential Calculator 2 (CPC2) (Kang et al., 2017; Kong et al., 2007), with default settings. Analyses using CPC2 evaluated transcripts according to peptide length, Fickett score (codon usage bias and nucleotide composition), predicted isoelectric point (pI) and Open reading frame (ORF) integrity. These features were then integrated by the CPC2 support vector machine model to calculate protein-coding status for each novel transcript.

### RNApysoforms isoform rendering

RNApysoforms (Aguzzoli Heberle et al., 2024) was used to generate models of isoform exon-intron structure and compare annotated transcripts with newly uncovered isoforms from the same gene. Inputs for these analyses included the reference GTF (GRCh38), transcript biotype annotations, extended transcript annotations from isoform discovery, and a defined list of isoforms of interest. This package was used exclusively to visualize transcript structure.

## Data Availability

The original contributions presented in the study are publicly available. Raw long-read scRNA-seq data generated and utilized for this study are publicly accessible in the NIH Sequence Read Archive (SRA) (accession no. SRR37807425, BioProject no. PRJNA1432415, BioSample no. SAMN56321669). A complete long-read PIPseq is available at protocols.io: https://www.protocols.io/view/long-read-pipseq-hrx2b57qf. Reference files, annotations, and ColabFold protein modeling are publicly available at Zenodo: http://doi.org/10.5281/zenodo.17341210. All code used in the preprocessing pipeline is publicly accessible at: https://github.com/UK-SBCoA-EbbertLab/single_cell_nextflow_pipeline. All code used for the analyses outside of the preprocessing pipeline is publicly accessible at: https://github.com/UK-SBCoA-EbbertLab/single_cell_analysis_PBMCs/tree/main. HG38 reference genome sequence is available at: https://ftp.ensembl.org/pub/release-113/fasta/homo_sapiens/cdna/. HG38 reference GFF3 ann otation is available at: https://ftp.ensembl.org/pub/release-113/gff3/homo_sapiens/. Transcript annotations utilized to determine the novelty of new transcripts and genes can be found at the following links: Glinos et al.7: https://storage.googleapis.com/gtex_analysis_v9/long_read_data/flair_filter_transcripts.gtf.gz. Leung et al.8: https://zenodo.org/record/7611814/preview/Cupcake_collapse.zip#tree_item12/HumanCTX.collapsed.gff. Heberle et al.9: https://doi.org/10.5281/zenodo.8180677.
