# Supplementary material for: Decoding the human PBMC isonome: isoform-level resolution with single-cell long-read transcriptomics
Source: Front Genet. 2026 May 28;17:1782221. doi: 10.3389/fgene.2026.1782221 (PMC13252915; doi:10.3389/fgene.2026.1782221)
Supplement: Supplementary file 2 [file DataSheet1.docx]

**Supplemental Information**

**Additional Cell Marker information:**

We identified T cells by the co-expression of the γ, δ, and ε subunits of the CD3 complex (*CD3G*, *CD3D*, *CD3E*), a major co-receptor involved in activation of both CD4+ T cells and CD8^+^ T cells^1^. NK cells were defined by the co-expression of surface-level marker *KLRF1* (NKp80)^2^ and marker for cytotoxicity, *GZMB*^1,2^, a major function of NK cells. *ITGAM* (CD11b)^3^, and *IL2RB* (CD122)^2^, are not NK-specific but reflect their status as innate lymphocytes that participate in cytokine signaling. Monocyte-derived cells were distinguished using a key myeloid lineage marker, *CD33*, an inhibitory marker enriched in monocyte-derived dendritic cells (*LILRB4*), and functional receptors for immune responses (*FCGR2A*/CD32A, and *CLEC7A*/Dectin-1); this mixture of disease-relevant monocyte and dendritic cell markers in the absence of markers of macrophages led us to categorize this cluster as monocyte-derived^4–7^. B cells were identified based on expression of cell-type-specific surface markers *CD22, CD79A*, *MS4A1* (CD20), and *CD19*. *CD79A* and *CD22* are key genes involved in B cell receptor signaling^1,8^, while *MS4A1* (CD20) and *CD19* are involved with B cell activation^1,9^. Finally, small numbers of Megakaryocytes, the major platelet producers, were identified by high expression of *ITGA2B* (CD41) and *GP1BA* *(CD42b)*, two surface receptors involved in platelet aggregation, while *MPL* regulates megakaryocyte growth and development of platelets^1,10,11^.

Memory T cells were recognized via expression of *TCF7*, a transcription factor associated with self-renewal and maintenance of memory function, as well as *CCR7* and *SELL* (L-selectin), two genes associated with connection to the lymphatic system that enable long-term immune surveillance. Effector CD8^+^ T cells were characterized by co-expression of *CD8A* and *CD8B*, which together encode for the CD8 co-receptor, a crucial protein involved in antigen recognition and T cell signaling. These cells were also classified by co-expression of *KLRB1*, a surface marker enriched in populations exhibiting cytotoxic activity as well as *GATA3* and *CCL5*, markers tied to effector T cell function. Effector CD4^+^ T cells expressed high levels of *CD4* as well as *IL2RA* (CD25) and TNF, which act as markers for T cell activation. These cells co-expressed transcription factors *AHR* and *GATA3*, tied to activation and cytokine signaling responses.

**Package Version Used:**

| **Software** | **Version** | **Source** |
| --- | --- | --- |
| adjustText | 1.3.0 | <https://github.com/Phlya/adjustText> ^12^ |
| anndata | 0.11.3 | <https://github.com/scverse/anndata> ^13,14^ |
| Bambu | 3.8.1 | <https://github.com/GoekeLab/bambu> ^15^ |
| bioservices | 1.12.1 | <https://github.com/cokelaer/bioservices>^16^ |
| Dorado | 7.2.13+fba8e8925 | <https://github.com/nanoporetech/dorado> |
| gffcompare | 0.12.6 | <https://github.com/gpertea/gffcompare> ^17^ |
| igraph | 0.11.6 | <https://github.com/igraph/igraph> ^13,14^ |
| Java (OpenJDK) | 17.0.13 | <https://github.com/microsoft/openjdk-jdk17u> |
| joblib | 1.4.2 | <https://github.com/joblib/joblib> |
| leidenalg | 0.10.2 | <https://github.com/vtraag/leidenalg> ^18^ |
| matplotlib | 3.10.1 | <https://github.com/matplotlib/matplotlib> ^19,20^ |
| Minimap2 | 2.28-r1209 | <https://github.com/lh3/minimap2> |
| MinKNOW | 23.11.4 | <https://github.com/nanoporetech/minknow_api> |
| muon | 0.1.7 | <https://github.com/KellerJordan/Muon> ^21,22^ |
| numpy | 2.1.3 | <https://github.com/numpy/numpy> ^23^ |
| os  (Linux kernel) | 4.18.0-305.10.2.el8_4.x86_64 |  |
| pandas | 2.2.3 | <https://github.com/pandas-dev/pandas> ^24^ |
| polars | 1.24.0 | <https://github.com/pola-rs/polars> ^25,26^ |
| PIPseeker | 02.01.04 |  |
| pybiomart | 0.2.0 | <https://github.com/jrderuiter/pybiomart> |
| pychopper | 2.7.10 | <https://github.com/epi2me-labs/pychopper> |
| Python | 3.10.13 | [http://www.python.org](https://web.archive.org/web/20161206043833/http:/www.python.org/) |
| SAMtools | 1.19.2 | <https://github.com/samtools/samtools> |
| scanpy | 1.11.0 | <https://github.com/scverse/scanpy> ^27–29^ |
| scikit-image | 0.25.2 | <https://github.com/scikit-image/scikit-image> ^30^ |
| scikit-learn | 1.5.2 | <https://github.com/scikit-learn/scikit-learn> ^31^ |
| scipy | 1.15.2 | <https://github.com/scipy/scipy> |
| scrublet | 0.2.3 | <https://github.com/swolock/scrublet> ^32^ |
| scvi-tools | 1.3.0 | <https://github.com/scverse/scvi-tools> ^33,34^ |
| seaborn | 0.13.2 | <https://github.com/mwaskom/seaborn> ^35^ |
| SoupX | 1.6.2 | https://github.com/constantAmateur/SoupX/tree/master^36^ |
| statsmodels | 0.14.2 | <https://github.com/statsmodels/statsmodels> |
| torch | 2.6.0 | <https://github.com/pytorch/pytorch> ^37^ |

**Materials**

1. **General Laboratory Equipment Required**

Cell Culture Hood with UV, Centrifuge with swinging rotor and/or adapters suitable for 15mL and 1mL tubes (*Thermo Scientific* #755007210, #75005743 (1mL adapter), #75005743 (15mL adapter)), 2000xg Benchtop mini centrifuge compatible with 1.5mL and 0.5mL tubes (*Benchmark Scientific #C1012*), PCR Thermocycler (ProFlex PCR System, 3x32-well: Applied Biosystems #4484073), Hemacytometer and 10X Microscope for cell counting, Invitrogen Qubit Flex Fluorometer (*ThermoFisher Scientific* #Q33327), Digital Vortex Mixer (*Thermo Scientific* #88882009), Brandon Ultrasonics CPXH Series Ultrasonic Cleaning Bath (*Fisher Scientific* #15-336-125), Femto Pulse System (*Agilent*, #M5330AA), DynaMag-2 Magnet (*Invitrogen* #12321D), Laboratory balance (mg) (*FisherScientific* #01-919-149), HulaMixer Sample Mixer (*Invitrogen* #BB3362792), Motorized Serological Pipette Controller (*BrandTech* #26333), micropipettes (*Eppendorf* #3123000063 (P1000), #3123000055 (P200), #3123000039 (P20), #3123000020 (P10)), PromethION 24 (*ONT,* #PRO-SEQ024), PromethION Data Acquisition Unit (*ONT, #PRO-PRCA100*)

1. **Materials and Reagents Needed for PBMC Isolation (optional)**

Blood Collection tube, BD Vacutainer® K2EDTA 10mL (*VWR* #BDAM367525), SepMate-50 (IVD) (*Stemcell Technologies*, #85450), Ficoll PAQUE Plus (*Millipore Sigma* #17-1440-020), Sterile 50-mL conical tubes (*Eppendorf* #0030122178), Serological Pipettes (*VWR* #75816-090 (25mL), *VWR* #75816-100) (10mL)), eBioscience 10X RBC Lysis Buffer (Multi-species) (*Invitrogen* #00-4300-54), Fetal Bovine Serum (FBS) (Corning #35-015-CV), Ultrapure 20X Phosphate Buffered Saline (PBS) pH 7.5 (VWR #E703-1L), Ultrapure H2O

1. **PIPseq Equipment Required**

The following equipment is included in the PIPseq T20 3’ Single Cell Starter Equipment Kit (*Fluent* #FBS-SCR-STKIT): PIPseq Vortex Mixer (*Fluent* #*FBS-SCR-DVM*), PIPseq Dry Bath with heated lid (*Fluent* #FBS-SCR-PDB), PIPseq Dry Bath 1.5mL block (*Fluent* #FB0002498), PIPseq 4-tube stand, blue, for 1.5mL tubes (*Fluent* #FB0004722), PIPseq Rotating vortex base assembly (*Fluent* #FB0002100).

1. **Plastic Consumables Required for Long-Read PIPseq**
   1. **PIPseq Plastics:** The following consumables are included within the PIPseq T20 3’ Single Cell Consumables Kit v4.0PLUS (*Fluent* #FBS-SCR-T20-4-V4.05-6): 1.5mL Safe-Lock PCR Clean tubes (*Eppendorf* #022363212), 0.2mL PCR 8-tube strip without Cap (*Greiner Bio-One*, #373270), PCR 8-Cap strips, domed cap (*Greiner Bio-One*, #373270), 3mL syringe, 1mL, G22 blunt bottom syringe needle.
   2. **Other Plastics:** Standard low-retention pipette tips (*Genesee Scientific* #24-430 (1000uL), #24-412 (200uL), #26-404 (20uL), #24-401 (10uL)), Wide-bore low-retention pipette tips (*Genesee Scientific* #22-428 (1000uL), #22-425 (200uL)), 10mL serological pipettes (*VWR* #75816-100), Sterile DNase-, RNase-, DNA-free 15mL tubes (*Eppendorf,* #0030122160), 0.2mL thin-walled PCR tubes (*Genesee Scientific* #24-705), 1.5mL DNA LoBind tubes (*Eppendorf*, #022431021), Qubit Flex Assay Tube Strips (*Invitrogen* *#Q33252*)
2. **Kits Needed for Long-Read PIPseq**
   1. **PIPseq Kits:** PIPseq T20 3’ Single Cell RNA Ambient Kit v4.0PLUS (*Fluent* #FBS-SCR-T20-4-V4.05-1), PIPseq T20 3’ Single Cell RNA 4ºC Kit v3.0 or 4.0 (*Fluent* #FBS-SCR-T20-4-V3&V4-2), PIPseq T20 3’ Single Cell RNA -20ºC Kit v4.0PLUS (*Fluent* #FBS-SCR-T20-4-V4.05-3), PIPseq T20 3’ Single Cell RNA -80ºC Kit v4.0PLUS (*Fluent* #FBS-SCR-T20-4-V4.05-4)
   2. **Quality Control Kits:** Qubit 1X dsDNA High Sensitivity Assay Kit (*ThermoFisher* #Q33231), Genomic DNA 165kb Kit (*Agilent*, #FP-1002-0275)
   3. **ONT Kits:** The following reagents were used from ONT PCR-cDNA Sequencing Kit V14 (ONT #SQK-PCS114): Rapid Adapter (RA), Adapter Buffer (ADB), Flow Cell Tether (FCT), Flow Cell Flush (FCF), Sequencing Buffer (SB), Library Beads (LIB), Elution Buffer (EB).
3. **Additional Reagents and Materials Needed for Long-Read PIPseq**
   1. **PIPseq:** SUPERase•In RNase Inhibitor (20U/uL) (*Invitrogen* # AM2696), 100% Ethanol molecular biology grade (*Millipore sigma* #EX0276-4), Tris-EDTA (TE) Buffer 1X solution pH 8.0 (*Fisher Bioreagents* #BP2473-1), IDTE (pH 8.0), Ultrapure water
   2. **Quality Control (QC):** Trypan Blue Solution (*PromoKine* #PK-CA902-1209), RNaseZap RNase Decontamination Solution (*Invitrogen,* #AM9782)
   3. **Oxford Nanopore Technologies (ONT) Sequencing Preparation:** 100% Ethanol molecular biology grade (*Millipore sigma* #EX0276-4), Tris Base (*Fisher Bioreagents* #BP152-1), HCl 5.0N (*VWR* #BDH7419-1), NaCl (*VWR* #0241-500G), 0.5M EDTA pH 8.0 *(Invitrogen* #AM9260G), Tris-EDTA (TE) Buffer 1X solution pH 8.0 (*Fisher Bioreagents* #BP2473-1), Ultrapure water, AMPure XP bead-based reagent (*Beckman* #A63880), M280 streptavidin 10ug/uL (*Invitrogen* #11205D), LongAmp Hot start Taq 2X Master Mix (*New England BioLabs* #M0533S), R10.4.1 flow cells (*ONT* #FLO-PRO114M)

**Supplemental Tables:**

| **Table S1. Unfiltered pseudo-bulk read metrics throughout preprocessing from PBMC samples collected across 3 flow cells per sample.** |
| --- |
| \|  \| **PBMC1** \| **PBMC2** \| \| --- \| --- \| --- \| \| N reads (total) \| 275,827,438 \| 215,987,216 \| \| N passed reads (ONT) \| 228,920,664 \| 163,153,766 \| \| N Reads After Pychopper \| 221,804,949 \| 157,166,409 \| \| N Reads After Barcode Correction \| 158,309,133 \| 103,099,471 \| \| N Reads After PIPseeker \| 153,038,291 \| 99,777,656 \| \| N trimmed and processed passed reads \| 153,038,291 \| 99,777,656 \| \| N reads aligned \| 135,302,551 \| 89,261,231 \| \| N reads aligned (MAPQ≥10) \| 121,867,484 \| 80,033,261 \| \| Reads mapping to positive strand (millions) \| 70.5 M \| 46.2 M \| \| Reads mapping to negative strand (millions) \| 51.4 M \| 33.8 M \| \| Non-splice reads (millions) \| 58.9 M \| 37.5 M \| \| Splice reads (millions) \| 63 M \| 42.4 M \| \| N50 FASTQ (mean across flow cells, nt) \| 658.67 \| 664.67 \| \| Median read length (nt) \| 476.67 \| 501.33 \| |

| **Table S2.** **Filtering criteria for cell quality.** |
| --- |
| \|  \| **Lower Threshold (≥)** \| **Upper Threshold (≤)** \| \| --- \| --- \| --- \| \| Mitochondrial Gene % \| N/A \| 15 \| \| Hemoglobin Gene % \| N/A \| 5 \| \| Total Counts \| 600 \| 11000 \| \| Unique Gene Count \| 250 \| 1800 \| \| Unique Isoform Count \| 350 \| 3000 \| |

| **Table S3. Proportion of genes and isoforms predicted to be zero-inflated by AutoZI Modeling.** | |
| --- | --- |
|  | Fraction |
| Predicted ZI Genes | 0.996 |
| Genes with average expression > 1.0 | 0.009 |
| Predicted ZI Genes with average expression > 1.0 | 0.575 |
| Predicted ZI Isoforms | 0.487 |
| Isoforms with Average Expression > 1.0 | 0.002 |
| Predicted ZI Genes with average expression > 1.0 | 0.565 |
| Fractions represent proportions of genes or isoforms meeting each criterion. Mean expression refers to average raw counts per feature across all cells. Values >1.0 indicate an average of at least 1 raw count per cell. | |

| **Table S4. Marker genes for cell-type and sub-cell-type cluster annotation.** | | |
| --- | --- | --- |
|  |  | Markers |
| T cells |  | CD3D+, CD3E+, CD3G+ |
|  | CD8+ Effector | CD8A+, CD8B+, GATA3, KLRB1+, CCL5+ |
|  | CD4+ Effector | CD4+, IL2RA+, GATA3+, AHR+, TNF+ |
|  | Memory | CCR7+, SELL+, TCF7+ |
|  | Effector-Memory Transition | ITGAE+, LEF1+ IL2RA+ CTLA4+, GATA3+, IL7R+, CD27+, TCF7+ |
| Natural Killer (NK) |  | GZMB+, KLRF1+, NCAM1+, ITGAM+, IL2RB+ |
| B cells |  | CD22+, CD79A+, MS4A1+, CD19+ |
| Monocyte-derived |  | FCGR2A+, CLEC7A+, CD33+, LILRB4+ |
| Megakaryocyte |  | GP1BA+, MPL+, ITGA2B+ |

| **Table S5. Cell-type-specific counts predicted for ambient RNA contamination.** | | | | | | | | | | |
| --- | --- | --- | --- | --- | --- | --- | --- | --- | --- | --- |
| Cell type | **Median** | **Median %** | **Mean** | **Mean %** | **Std** | **Std %** | **Min.** | **Min. %** | **Max.** | **Max. %** |
| B cells | 21.23 | 1.04 | 28.899 | 1.002 | 27.35 | 0.648 | -22.53 | -2.279 | 174.99 | 3.774 |
| Monocyte-derived | 24.52 | 1.087 | 27.653 | 1.026 | 23.923 | 0.817 | -24.01 | -2.388 | 133.68 | 4.144 |
| CD8^+^  T cells | 21.52 | 1.049 | 29.75 | 1.007 | 27.59 | 0.597 | -22.1 | -1.9 | 167.23 | 3.852 |
| CD4^+^  T cells | 27.06 | 1.064 | 36.973 | 1.015 | 31.648 | 0.521 | -22.07 | -2.64 | 176.95 | 3.697 |
| Memory  T cells | 19.99 | 1.042 | 26.758 | 1.015 | 25.277 | 0.58 | -19.84 | -1.904 | 202.25 | 4.396 |
| NK cells | 19.6 | 1.064 | 26.223 | 1.008 | 25.042 | 0.718 | -24.74 | -2.439 | 161.72 | 4.464 |
| Transition  T cells | 21.43 | 1.117 | 23.929 | 1.112 | 17.908 | 0.563 | -6.18 | -0.655 | 148.979 | 3.894 |
| Megakary-ocytes | 13.135 | 0.915 | 17.679 | 0.962 | 17.642 | 0.775 | -9.97 | -0.927 | 93.21 | 2.611 |

| **Table S6. Protein sequences used for AlphaFold modeling, retrieved from Uniprot (v2025_03)**^38^**.** | | |
| --- | --- | --- |
|  |  | **Sequence:** |
| ***GZMB-201***  *(ENST00000216341)* | Canonical protein-coding | **Known Protein Sequence (UniProt: P10144)** |
|  |  | MQPILLLLAFLLLPRADAGEIIGGHEAKPHSRPYMAYLMIWDQKSLKRCGGFLIRDDFVLTAAHCWGSSINVTLGAHNIKEQEPTQQFIPVKRPIPHPAYNPKNFSNDIMLLQLERKAKRTRAVQPLRLPSNKAQVKPGQTCSVAGWGQTAPLGKHSHTLQEVKMTVQEDRKCESDLRHYYDSTIELCVGDPEIKKTSFKGDSGGPLVCNKVAQGIVSYGRNNGMPPRACTKVSSFVHWIKKTMKRY |
| ***GZMB-204***  *(ENST00000216341)* | Protein-coding | **Known Protein Sequence (UniProt: E9PRD7)** |
|  |  | MQPILLLLAFLLLPRADAGEIIGGHEAKPHSRPYMAYLMIWDQKSLKRCGGFLIRDDFVLTAAHCWGSWRERPSGPELCSPSGYLATRPR |
| **CD3G-206**  (ENST00000532917) | Canonical protein-coding | **Known Protein Sequence (UniProt: P09693)** |
|  |  | MEQGKGLAVLILAIILLQGTLAQSIKGNHLVKVYDYQEDGSVLLTCDAEAKNITWFKDGKMIGFLTEDKKKWNLGSNAKDPRGMYQCKGSQNKSKPLQVYYRMCQNCIELNAATISGFLFAEIVSIFVLAVGVYFIAGQDGVRQSRASDKQTLLPNDQLYQPLKDREDDQYSHLQGNQLRRN |
| **CD3G-202**  (ENST00000392883) | Protein-coding | **Known Protein Sequence (UniProt: A8MUH3)** |
|  |  | MEQGKGLAVLILAIILLQGTLAQSIKGNHLVKVYDYQEDGSVLLTCDAEAKNITWFKDGKMIGFLTEDKKKWNLGSNAKDPRGMYQCKGSQNKSKPLQVYYRTSDKQTLLPNDQLYQPLKDREDDQYSHLQGNQLRRN |

| **Table S7. Study Protocol Quality Control (QC) metrics.** |
| --- |
| \|  \| PBMC1 \| PBMC2 \| \| --- \| --- \| --- \| \| PIPseq cDNA Yield (ng/µL) \| 10.08 \| 17.95 \| \| Peak bp average after PIPseq Protocol \| 1064.5 \| 956 \| \| ONT cDNA Yield (ng/µL) (input = 10ng) \| 20.90 \| 22.20 \| \| Peak bp average after ONT Protocol \| 1189.5 \| 1142.5 \| \| A260/A280 ratio after ONT Protocol (ng/µL) \| 1.72 \| 2.18 \| |

| **Table S8. Summary Statistics After Filtering.** |
| --- |
| \|  \|  \| PBMC1 \| PBMC2 \| \| --- \| --- \| --- \| --- \| \| Total Gene counts per cell \| Min \| 800.00 \| 800.00 \| \| Median \| 4558.5 \| 1760.00 \| \| Mean \| 4640.38 \| 1906.00 \| \| Max \| 9999.0 \| 9707.00 \| \| Transcripts per Gene \| Min \| 1.63 \| 1.45 \| \| Median \| 4.48 \| 2.96 \| \| Mean \| 4.51 \| 3.00 \| \| Max \| 8.59 \| 6.45 \| \| Unique Genes per Cell \| Min \| 350.00 \| 350.00 \| \| Median \| 1003.00 \| 596.00 \| \| Mean \| 997.33 \| 630.06 \| \| Max \| 1700.00 \| 1694.0 \| \| Mitochondrial Percentage \| Min \| 0.00 \| 0.00 \| \| Median \| 7.34 \| 7.15 \| \| Mean \| 7.63 \| 7.50 \| \| Max \| 15.00 \| 14.99 \| \| Total Isoform Counts per cell \| Min \| 800.11 \| 800.01 \| \| Median \| 4405.01 \| 1689.94 \| \| Mean \| 4504.40 \| 1831.13 \| \| Max \| 9991.86 \| 9227.28 \| \| Reads per Isoform per Cell \| Min \| 0.84 \| 0.70 \| \| Median \| 2.72 \| 1.82 \| \| Mean \| 2.76 \| 1.84 \| \| Max \| 5.49 \| 3.77 \| \| Unique Isoforms per cell \| Min \| 501 \| 500 \| \| Median \| 1596.0 \| 936.0 \| \| Mean \| 1603.89 \| 1004.75 \| \| Max \| 2995 \| 2992 \| |

**Supplemental Figures**

| 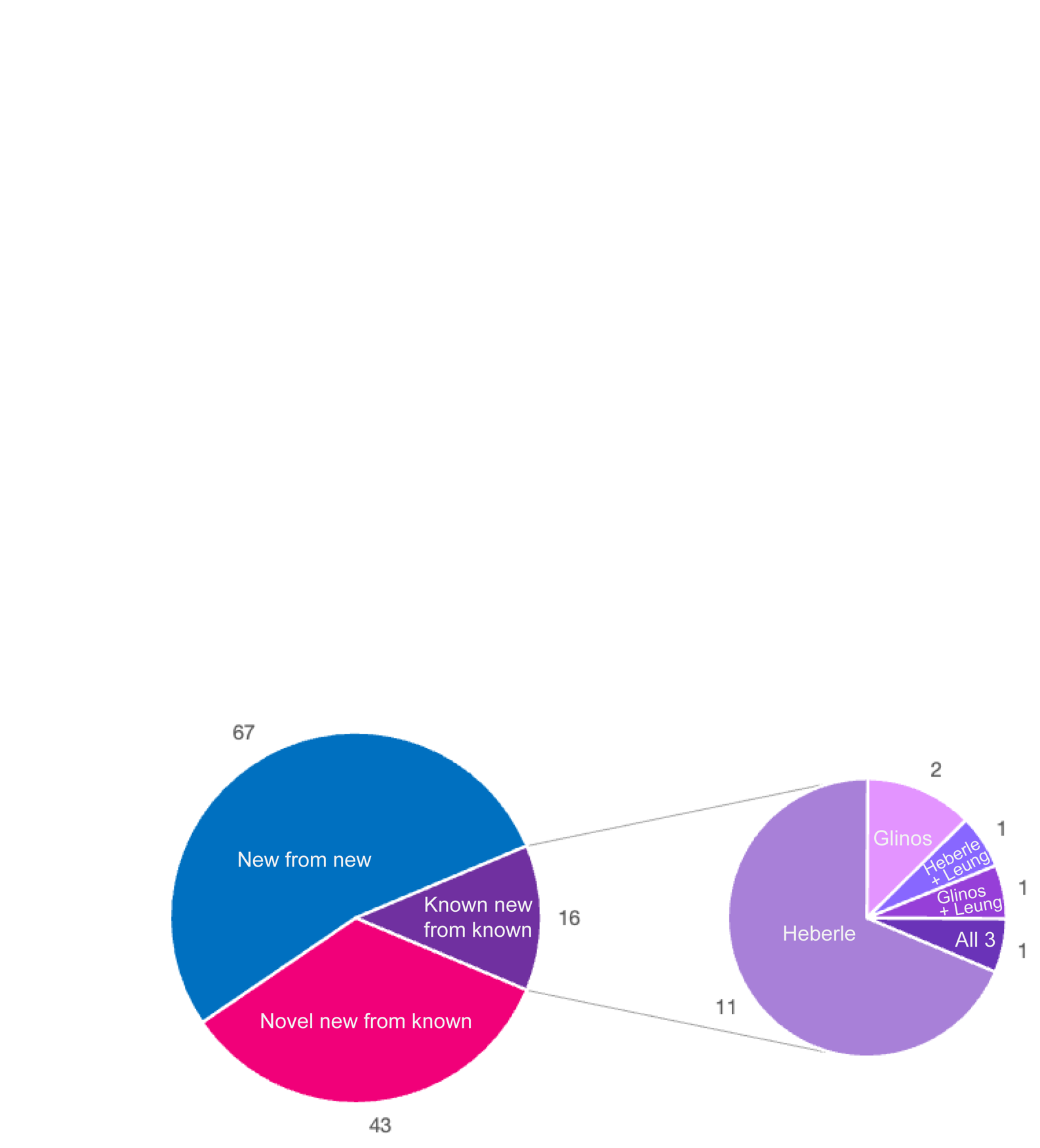 |
| --- |
| **Figure S1. Incidence of newly identified isoforms from known and new genes in existing datasets.** Pie charts showing overlap of new transcripts from this study. Of the 16 isoforms from our “known new from known” list of isoforms, the highest majority (11) were from Heberle *et al.*^39^*,* followed by Glinos *et al*.^40^ (2), and Leung *et al.*^41^ A single isoform (BambuTx85 from *MYL5*) was identified in all three datasets*.* |

|  |
| --- |
| **Figure S2. Distribution of isoform length in PBMC datasets.** Histogram showing distribution of transcript lengths (in nucleotides) for all isoforms detected. The median is 1737nt, as highlighted by the red line. |

| 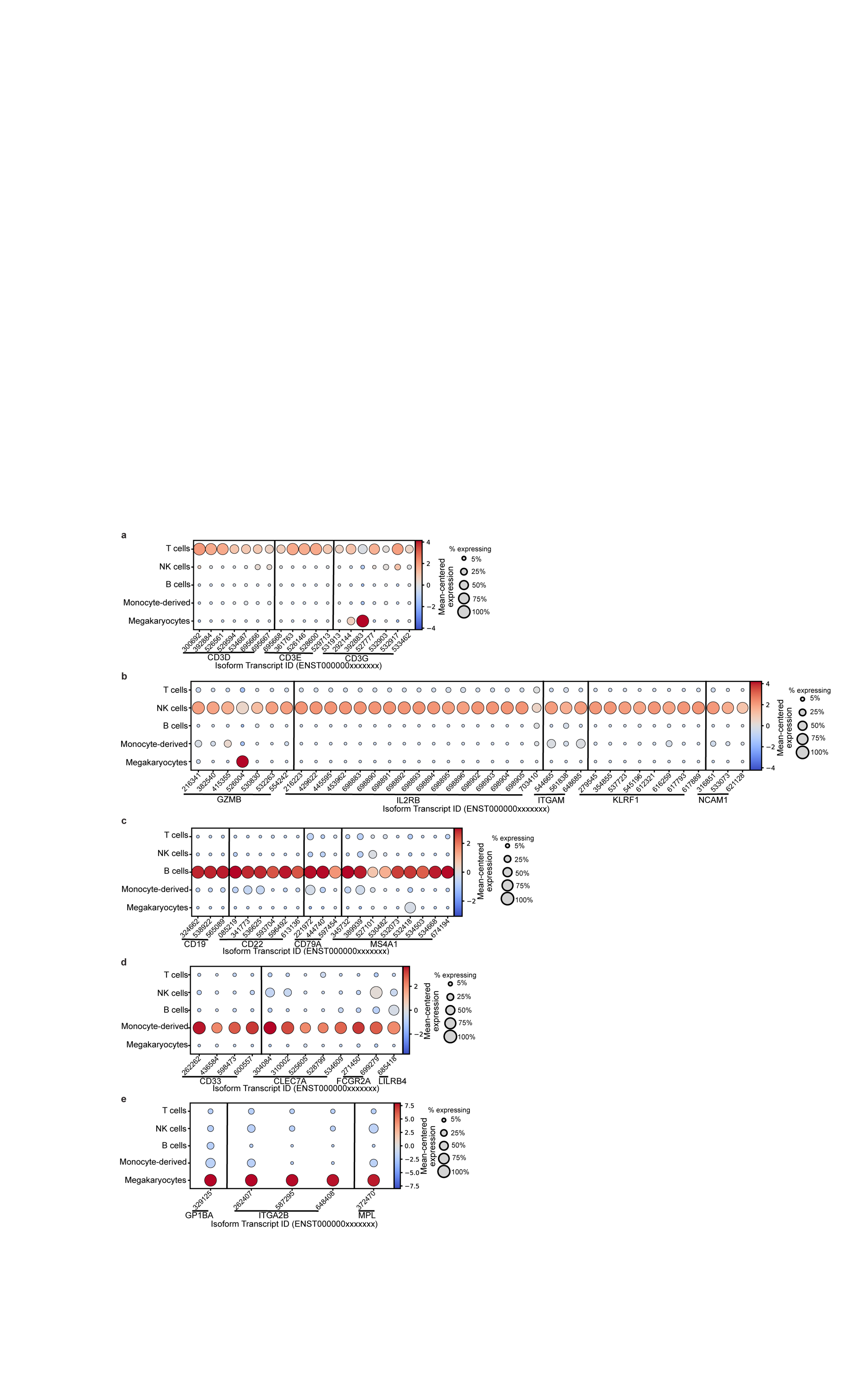 |
| --- |
| **Ƒigure S3. Cell-type marker gene isoform expression across major PBMC cell types**. (a-e) Dotplots showing normalized expression (Mean-centered, color scale) and percentage of cells per cluster expressing each transcript (dot size) across five major PBMC cell-types: (a) T cells (*CD3D, CD3E, CD3G*), (b) Natural Killer (NK) cells (*GZMB, IL2RB, ITGAM, KLRF1, NCAM1),* (c) B cells (*CD19, CD22, CD79A, MS4A1*), (d) Monocyte-derived cells (*CD33, CLEC7A, FCGR2A, LILRB4*), and (e) Megakaryocytes (*GP1BA, ITGA2B, MPL*). Isoform transcript identifiers correspond to Ensembl ID, starting with “ENST00000” and showing the last 6 digits on the y-axis of the corresponding point. |

| 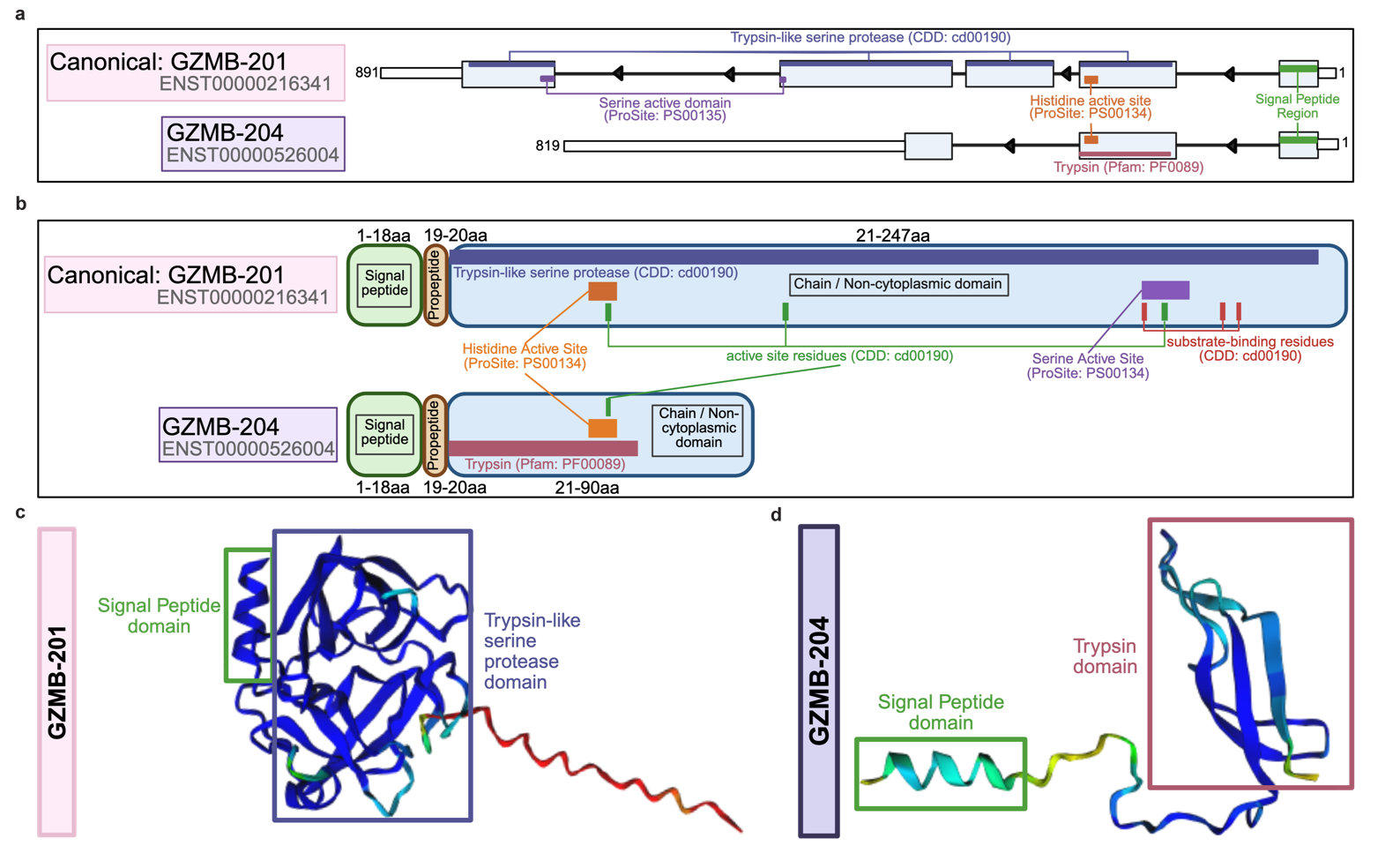 |
| --- |
| **Figure S4**. **Protein-coding region significance and predicted structures of *GZMB* isoforms.**  **(a)** GZMB-201 and GZMB-204 RNA isoform structure on the negative strand, based on results from SAPFIR^42^. Both isoforms encode an N-terminal signal peptide region, while GZMB-201 contains the entire trypsin-like serine protease domain with histidine and serine active sites. GZMB-204 lacks exons 2 and 5, removing the serine active domain and changing the specificity of the trypsin domain. **(b)** Domain architecture of the proteins resulting from GZMB-201 and GZMB-204 according to InterProScan^43,44^, including histidine and serine active sites and conserved residues. GZMB-204 exhibits loss of active site and substrate-binding residues in the mature functional polypeptide (chain region)*.* **(c-d)** Predicted protein structure of GZMB-201 **(c)** and GZMB-204, missing the serine active site and reducing domain specificity **(d)**, modeled by AlphaFold, with confidence scores colored from blue (high) to red (low). AlphaFold output files for these structures including .PDB of the final structure can be found on Zenodo^45^, as described in **Data and Code Availability**. GZMB-204 exhibits the loss of protease domain integrity. *Created in BioRender. Ebbert, M. (2026)* [*https://BioRender.com/q8mh9zl*](https://biorender.com/q8mh9zl) |

| 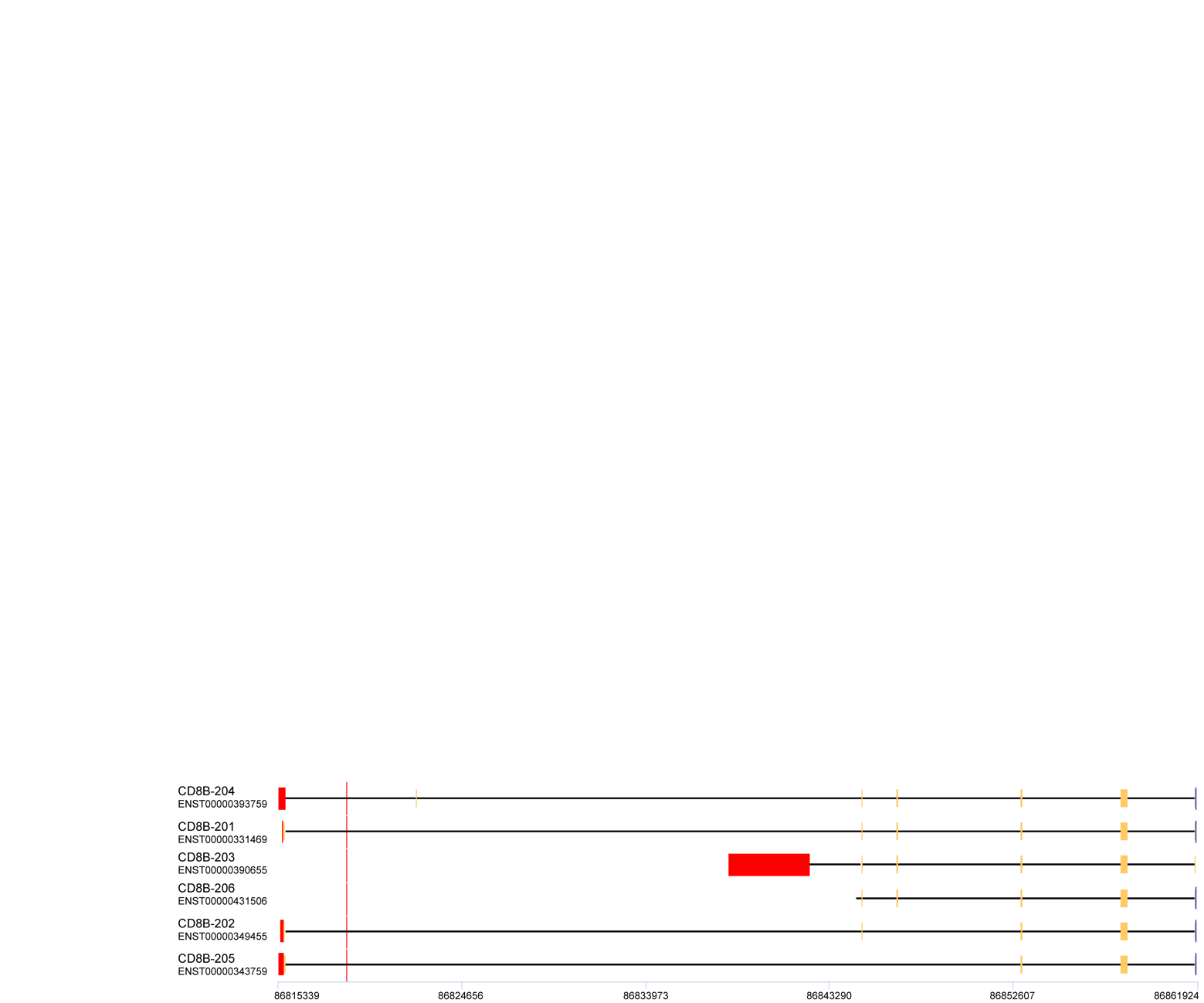 |
| --- |
| **Ƒigure S5. Alternative untranslated region (UTR) usage among *CD8B* isoforms as visualized by UTRdb**^46^**.** Screenshot from UTRdb showing predicted 3’ (red) and 5’UTR (blue) segments, with coding exons shown as yellow boxes. Each horizontal track represents a distinct isoform of CD8B, with black lines representing introns. |

| 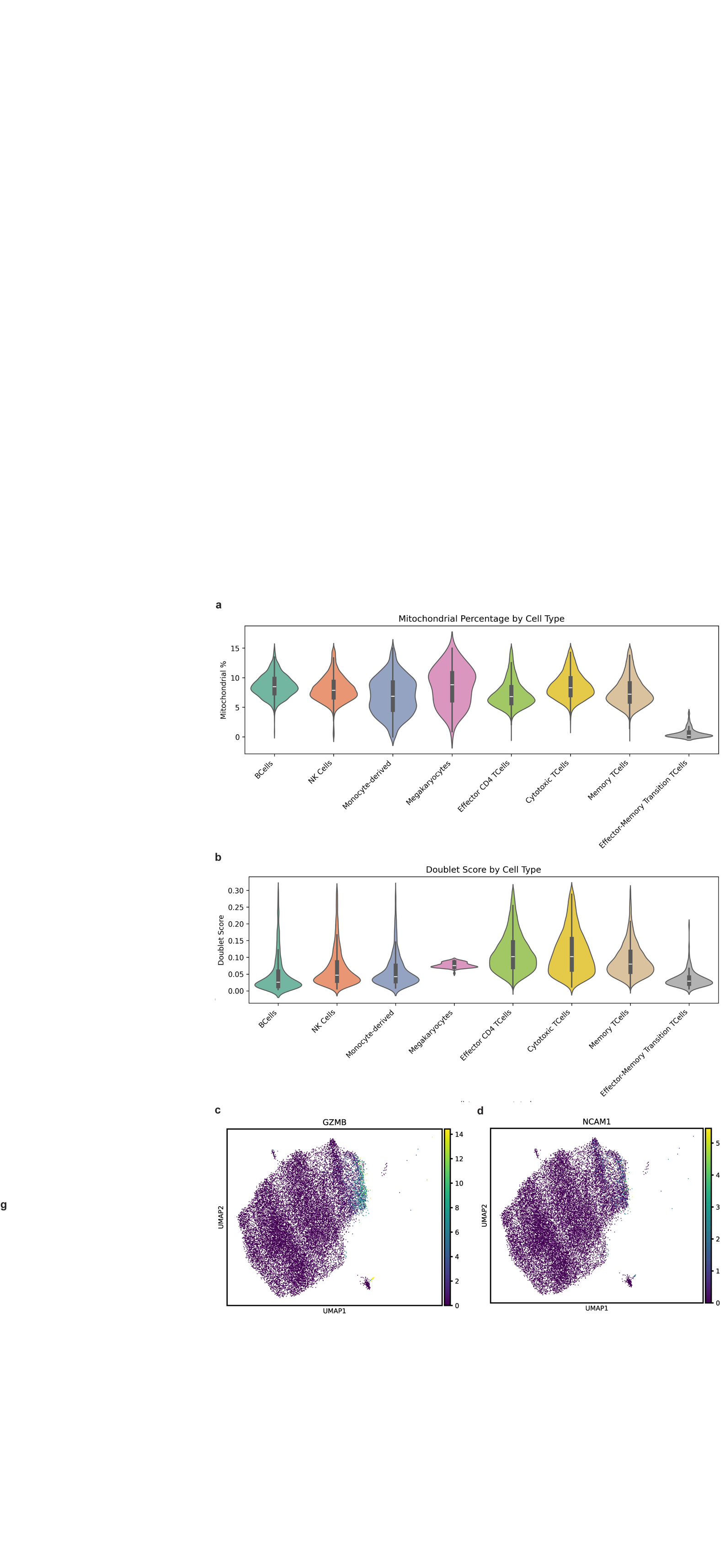 |
| --- |
| **Ƒigure S6. Quality control metrics confirm effector-memory transition T cells form a distinct cluster from NK-T cells. (a)** Distribution by cell-type of mitochondrial read percentage per cell. **(b)** Distribution by cell-type of doublet score as calculated by Scrublet^32^ python package. **(c)** UMAP colored by GZMB expression in T cell datasets. **(d)** UMAP colored by NCAM1 expression in T cell datasets. |

| 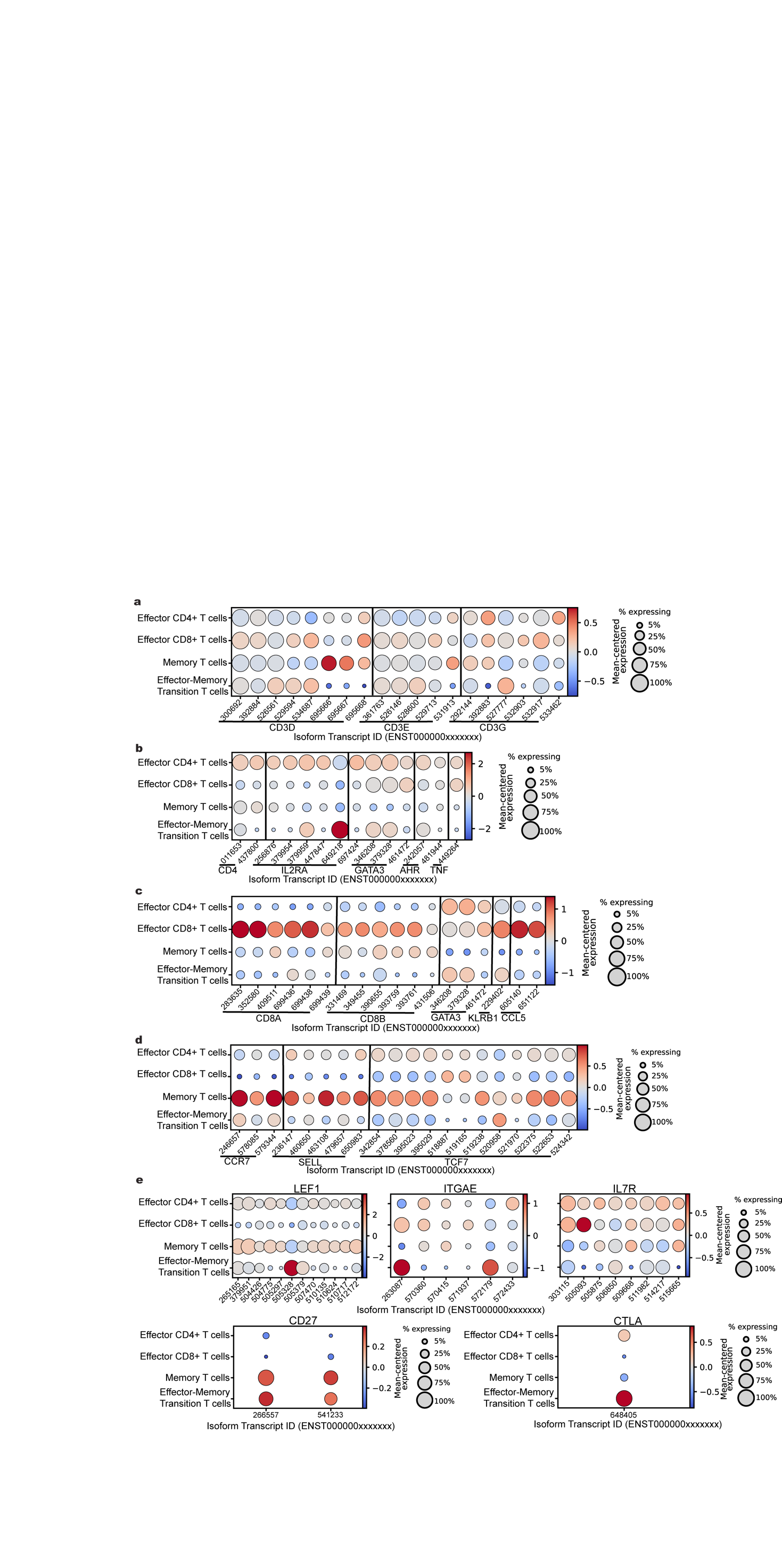 |
| --- |
| **Ƒigure S7. Isoform-specific expression patterns among T cell subtypes. (a-e)** Dot plots showing all isoforms from marker genes expressed in our dataset and their respective normalized expression relative to mean expression (color scale) and percentage of cells expressing that isoform (dot size), across all T cell subtypes. Isoform transcript identifiers correspond to Ensembl ID, starting with “ENST00000” and showing the last 6 digits on the y-axis of the corresponding point. Isoforms express enrichment of marker genes for **(a)** general T cell markers (*CD3D, CD3E, CD3G*), **(b)** Effector CD4^+^ T cells (*CD4, IL2RA, GATA3, AHR, TNF*), **(c)** Effector CD8^+^ T cells (*CD8A, CD8B, GATA3, KLRB1, CCL5*), **(d)** Memory T cells (*CCR7, SELL, TCF7*), **(e)** Effector-Memory Transition T cells (*LEF1, ITGAE, IL7R, CD27, CTLA*) |

**Supplemental References**

1. Oelen, R. *et al.* Single-cell RNA-sequencing of peripheral blood mononuclear cells reveals widespread, context-specific gene expression regulation upon pathogenic exposure. *Nat Commun* **13**, 3267 (2022).

2. Freud, A. G., Mundy-Bosse, B. L., Yu, J. & Caligiuri, M. A. The Broad Spectrum of Human Natural Killer Cell Diversity. *Immunity* **47**, 820–833 (2017).

3. Fu, B., Tian, Z. & Wei, H. Subsets of human natural killer cells and their regulatory effects. *Immunology* **141**, 483–489 (2014).

4. Yao, J. *et al.* Genetic landscape and immune mechanism of monocytes associated with the progression of acute-on-chronic liver failure. *Hepatol Int* **17**, 676–688 (2023).

5. Aghaei, M. *et al.* Genetic variants of dectin-1 and their antifungal immunity impact in hematologic malignancies: A comprehensive systematic review. *Curr Res Transl Med* **72**, 103460 (2024).

6. Liu, J. *et al.* LILRB4, from the immune system to the disease target. *Am J Transl Res* **12**, 3149–3166 (2020).

7. Kapellos, T. S. *et al.* Human Monocyte Subsets and Phenotypes in Major Chronic Inflammatory Diseases. *Front Immunol* **10**, 2035 (2019).

8. Clark, E. A. & Giltiay, N. V. CD22: A Regulator of Innate and Adaptive B Cell Responses and Autoimmunity. *Front Immunol* **9**, 2235 (2018).

9. Depoil, D. *et al.* CD19 is essential for B cell activation by promoting B cell receptor-antigen microcluster formation in response to membrane-bound ligand. *Nat Immunol* **9**, 63–72 (2008).

10. Ng, A. P. *et al.* Mpl expression on megakaryocytes and platelets is dispensable for thrombopoiesis but essential to prevent myeloproliferation. *Proc Natl Acad Sci U S A* **111**, 5884–5889 (2014).

11. Guo, K., Machlus, K. R. & Camacho, V. The many faces of the megakaryocytes and their biological implications. *Curr Opin Hematol* **31**, 1–5 (2024).

12. Ilya Flyamer *et al.* Phlya/adjustText: 1.3.0. Zenodo https://doi.org/10.5281/ZENODO.14019059 (2024).

13. Virshup, I. *et al.* The scverse project provides a computational ecosystem for single-cell omics data analysis. *Nat Biotechnol* **41**, 604–606 (2023).

14. Virshup, I., Rybakov, S., Theis, F. J., Angerer, P. & Wolf, F. A. anndata: Access and store annotated datamatrices. *JOSS* **9**, 4371 (2024).

15. Chen, Y. *et al.* Context-aware transcript quantification from long-read RNA-seq data with Bambu. *Nat Methods* **20**, 1187–1195 (2023).

16. Cokelaer, T., Pultz, D., Harder, L. M., Serra-Musach, J. & Saez-Rodriguez, J. BioServices: a common Python package to access biological Web Services programmatically. *Bioinformatics* **29**, 3241–3242 (2013).

17. Pertea, G. & Pertea, M. GFF Utilities: GffRead and GffCompare. *F1000Res* **9**, 304 (2020).

18. Traag, V. A., Waltman, L. & Van Eck, N. J. From Louvain to Leiden: guaranteeing well-connected communities. *Sci Rep* **9**, 5233 (2019).

19. The Matplotlib Development Team. Matplotlib: Visualization with Python. Zenodo https://doi.org/10.5281/ZENODO.14940554 (2025).

20. The Matplotlib Development Team. Matplotlib: Visualization with Python. Zenodo https://doi.org/10.5281/ZENODO.592536 (2025).

21. Jordan, K. *et al.* Muon: An optimizer for hidden layers in neural networks. (2024).

22. Li, H. Minimap2: pairwise alignment for nucleotide sequences. *Bioinformatics* **34**, 3094–3100 (2018).

23. Harris, C. R. *et al.* Array programming with NumPy. *Nature* **585**, 357–362 (2020).

24. The pandas development team. pandas-dev/pandas: Pandas. Zenodo https://doi.org/10.5281/ZENODO.17229934 (2025).

25. Ritchie Vink *et al.* pola-rs/polars: Python Polars 1.29.0. Zenodo https://doi.org/10.5281/ZENODO.15313193 (2025).

26. Polars Developers. Polars: A Fast DataFrame Library for Python and Rust. (2025).

27. Virshup, I. *et al.* The scverse project provides a computational ecosystem for single-cell omics data analysis. *Nat Biotechnol* **41**, 604–606 (2023).

28. Wolf, F. A., Angerer, P. & Theis, F. J. SCANPY: large-scale single-cell gene expression data analysis. *Genome Biol* **19**, 15 (2018).

29. Li, H. *et al.* The Sequence Alignment/Map format and SAMtools. *Bioinformatics* **25**, 2078–2079 (2009).

30. Van Der Walt, S. *et al.* scikit-image: image processing in Python. *PeerJ* **2**, e453 (2014).

31. Pedregosa, F. *et al.* Scikit-learn: Machine Learning in Python. *Journal of Machine Learning Research* **12**, 2825–2830 (2011).

32. Wolock, S. L., Lopez, R. & Klein, A. M. Scrublet: Computational Identification of Cell Doublets in Single-Cell Transcriptomic Data. *Cell Syst* **8**, 281-291.e9 (2019).

33. Clivio, O. *et al.* Detecting Zero-Inflated Genes in Single-Cell Transcriptomics Data. Preprint at https://doi.org/10.1101/794875 (2019).

34. Gayoso, A. *et al.* A Python library for probabilistic analysis of single-cell omics data. *Nat Biotechnol* **40**, 163–166 (2022).

35. Waskom, M. L. seaborn: statistical data visualization. *Journal of Open Source Software* **6**, 3021 (2021).

36. Young, M. D. & Behjati, S. SoupX removes ambient RNA contamination from droplet-based single-cell RNA sequencing data. *GigaScience* **9**, giaa151 (2020).

37. Paszke, A. *et al.* PyTorch: An Imperative Style, High-Performance Deep Learning Library. Preprint at https://doi.org/10.48550/ARXIV.1912.01703 (2019).

38. The UniProt Consortium *et al.* UniProt: the Universal Protein Knowledgebase in 2025. *Nucleic Acids Research* **53**, D609–D617 (2025).

39. Heberle, B. A. *et al.* *Using Deep Long-Read RNAseq in Alzheimer’s Disease Brain to Assess Medical Relevance of RNA Isoform Diversity*. http://biorxiv.org/lookup/doi/10.1101/2023.08.06.552162 (2023) doi:10.1101/2023.08.06.552162.

40. Glinos, D. A. *et al.* Transcriptome variation in human tissues revealed by long-read sequencing. *Nature* **608**, 353–359 (2022).

41. Leung, S. K. *et al.* Full-length transcript sequencing of human and mouse cerebral cortex identifies widespread isoform diversity and alternative splicing. *Cell Rep* **37**, 110022 (2021).

42. Zhou, D., Tran, Y., Abou Elela, S. & Scott, M. S. SAPFIR: A webserver for the identification of alternative protein features. *BMC Bioinformatics* **23**, 250 (2022).

43. Jones, P. *et al.* InterProScan 5: genome-scale protein function classification. *Bioinformatics* **30**, 1236–1240 (2014).

44. Blum, M. *et al.* InterPro: the protein sequence classification resource in 2025. *Nucleic Acids Res* **53**, D444–D456 (2025).

45. Ebbert, M., Doyle, P. & Page, M. Ebbert_lab_PBMC_long_read_single_cell_project. Zenodo https://doi.org/10.5281/ZENODO.17341210 (2025).

46. Lo Giudice, C. *et al.* UTRdb 2.0: a comprehensive, expert curated catalog of eukaryotic mRNAs untranslated regions. *Nucleic Acids Res* **51**, D337–D344 (2023).
